# SFN Enhanced the Radiosensitivity of Cervical Cancer Cells via Activating LATS2 and Blocking Rad51/MDC1 Recruitment to DNA Damage Site

**DOI:** 10.3390/cancers14081872

**Published:** 2022-04-08

**Authors:** Shiyu Wang, Yanan Wang, Xiangnan Liu, Yongbin Yang, Sufang Wu, Yuan Liu

**Affiliations:** Department of Obstetrics and Gynecology, Shanghai General Hospital, Shanghai Jiao Tong University School of Medicine, Shanghai 201620, China; wangshiyu1108@sina.com (S.W.); wangyn0929@126.com (Y.W.); liuxiangnan2022@163.com (X.L.); yybvsywz@163.com (Y.Y.)

**Keywords:** sulforaphane (SFN), LATS2, ionizing radiation (IR), DNA double strand breaks (DSB), DNA damage repair (DDR)

## Abstract

**Simple Summary:**

Radiotherapy is the main treatment for cervical cancer patients in advanced stages. However a considerable number of patients are not sensitive to radiotherapy. Dysregulation of DNA double-strand break (DSB) repair is characteristic of cancer cells in a radiotherapy-resistance state. The aim of this study is to explore Sulforaphane (SFN) downstream target and the radiotherapy sensitization mechanism in cervical cancer. We identified SFN as cervical cancer cells radiotherapy sensitizer and LATS2 served as a downstream target of SFN treatment. SFN treatment resulted in the inhibition of the homologous recombination (HR) pathway, and LATS2 has an indispensable contribution to this SFN-facilitated radiotherapy sensitization.

**Abstract:**

Background: Sulforaphane (SFN) is one kind of phytochemical anticancer drug. It inhibits cancer cell proliferation and promotes cell apoptosis while the mechanism behind is still uncertain. We aimed to explore its downstream target and the radiotherapy sensitization mechanism in cervical cancer. Methods: We treated established cervical cancer cells line (SiHa, HeLa, C33A) with SFN followed by irradiation, and explored its survival, apoptosis, and DNA damage repair in vitro and validated the radiosensitivity of SFN treatment in vivo. We conducted mRNA sequencing to identify differentially expressed mRNAs after SFN treatment. We further investigated SFN downstream target and its involvement in DNA damage repair under irradiation. Results: We found that SFN inhibited the survival of cervical cancer cells under radiotherapy treatment in vitro and prolonged the survival period after radiotherapy in the mouse tumorigenic model. SFN increased the protein expression of LATS2 and promoted apoptosis of cervical cancer cells. Overexpressed LATS2 decreased the cellular survival rate of cervical cancer cells. Additionally, SFN treatment and LATS2 overexpression prevented MDC1 and Rad51 from accumulating in the nucleus in cervical cancer cells after being exposed to ionized radiation. LATS2 loss intervened with SFN-alleviated RAD51 and MDC1 nucleus accumulation and resumed the repairment of DNA damage. Conclusion: We identified SFN as cervical cancer cells radiotherapy sensitizer and LATS2 served as a downstream target of SFN treatment. SFN treatment resulted in the inhibition of the homologous recombination (HR) pathway, and LATS2 has an indispensable contribution to this SFN-facilitated radiotherapy sensitization.

## 1. Introduction

Cervical cancer is the fourth most commonly found cancer in women worldwide and the second in less developed regions [1,2]. HPV oncoprotein is the primary etiological cause of cervical cancer by prolonging cell cycle progression, delaying cell differentiation, and inhibiting apoptosis in host keratinocyte cells. Additionally, HPV leads to acquisition of host genetic mutation and mediates the evasion of the host immune response [3]. Since the application of HPV vaccines and PAP smear, there has been substantial decreasing incidence of cervical cancer; although, the latency period from HPV infection, to intraepithelial lesion, to the onset of carcinoma remains an issue [4,5,6]. According to relevant statistics, 13% of patients with cervical cancer are diagnosed in advanced stage, resulting in a decrease in the rate of patient survival [7]. Surgery and concurrent chemoradiotherapy (CCRT) are the standard treatments for cervical cancer. According to NCCN clinical practice guideline 2020, CCRT is generally recommended to be the primary treatment of choice for stages higher than IB2 and IIA1, and for patients who are not candidates for hysterectomy [8]. Radiotherapy reduces the recurrence of cervical cancer by 13% [9]. Thus, radiotherapy is the main treatment for patients in advanced stages. Cervical squamous cell carcinoma is the most common pathological type accounting for approximately 70% of all cervical cancers, and it is moderately sensitive to radiation. However, a considerable number of patients are not sensitive to radiotherapy, which remains the biggest obstacle to the treatment of cervical cancer.

The responses of cancer cells to radiotherapy vary in their respective resistance mechanisms. There are several mechanisms explaining the irradiation resistance. Firstly, direct ionization or indirect OH radicals could damage a base, resulting in DNA single- or double-strand break or produce a DNA crosslink, among which, DNA double-strand break (DSB) is the major type. However, these lesions are essentially repaired by the DNA damage repair mechanism in cancer cells including two pathways—non-homologous end-joining (NHEJ) and homologous recombination (HR). Additionally, radioresistance can be impacted by the lack of oxygen, cell cycle status, apoptosis, growth factors, and stem cells, etc. [10]. Among them, DSB repair is the most important. Dysregulation of DSB repair is characteristic of cancer cells in a radiotherapy-resistance state and explains broad resistance to other therapeutic treatments. Studies have reported radiosensitization targets including suppression of DSB repair, restriction of survival pathways, induction of cell death pathways, and reactivation of p53 [11]; meanwhile, effective and tumor-cell-distinguishable radiosensitization therapy remain to be found.

Sulforaphane (SFN, 1-isothiocyanato-4(R)-methylsulfinylbutane) is a thiocyanate extracted from cruciferous vegetables, specifically broccoli [12]. Previous studies showed that the payload of glucoraphanin is the precursor of sulforaphane and protect against various types of cancer. SFN may decrease the risk of cardiovascular disease and help in autism and osteoporosis [13]. As for oncobiology and tumorigenicity, SFN acts on several hallmarks of cancer including activation the transcriptional factor Nrf2 and the following induction of phase II detoxification enzymes. SFN also affects signal transduction pathways involved in growth regulation and acts as proapoptotic agent and an angiogenesis and metastases regulator in a dose-dependent manner [14]. Although SFN has been proposed to regulate the metabolism of carcinogens, the underlying mechanism remains elusive. SFN processed chemopreventive and chemotherapeutic potentiality but clinical trials about SFN have been limited.

In the current study, we aimed to explore the antitumor role of SFN in cervical cancer and investigate its downstream target and the radiotherapy sensitization mechanism. Our results indicated that SFN could enhance the expression of LATS2 and exert irradiation sensitization in cervical cancer cells by suppressing DNA double-strand break repair, specifically by inhibiting Rad51 and MDC1 nuclear accumulation. Our data helped to better explain the anticancer role of SFN and validate a cervical cancer radiosensitizer. We also identified the regulatory role for LATS2 in HR repair and our data suggested that SFN could be an appealing target to therapeutically induce DNA breaks and increase the sensitivity of cervical cancer cells to radiotherapy.

## 2. Materials and Methods

### 2.1. Reagents and Antibodies

Sulforaphane (>95% pure) was purchased from Pioneer Herb Industrial Co. Ltd. (Shanghai, China). For the antibodies, mouse monoclonal anti-cleaved PARP, rabbit monoclonal anti-cleaved caspase-3, and rabbit monoclonal anti-γ-H2AX monoclonal antibodies (7631) were purchased from Cell Signaling Technology (CST, Danvers, MA, USA). Rabbit monoclonal anti-Rad51 antibodies were purchased from Abcam Biotechnology (Danvers, MA, USA). Anti-LATS2 was from Proteintech (Ag28221). Anti-MDC1 antibody was from Abcam (Danvers, MA, USA, ab241048). Anti-Tubulin antibody, and CoraLite 594-conjugated goat anti-rabbit IgG secondary antibody were purchased from Proteintech (10694).

### 2.2. Cell Culture and Reagents

All cells were cultured in a 5% CO_2_ humidified incubator at 37 °C. DMEM F-12 (Gibco) with 10% fetal bovine serum (FBS; Gibco), 100 U/mL penicillin, sodium pyruvate, and L-glutamin. The human cervical carcinoma lines HPV16(+) SiHa, HPV18(+) HeLa, and HPV (−) C33A were purchased from ATCC. All cell lines used in this study were verified to be mycoplasma-negative cells. The cell lines were stocked in the laboratory of Dr. Sufang Wu for 2 years, and no further authentication was performed.

### 2.3. Ionizing Radiation

γ-irradiation was performed at the clinical transformation research center, Shanghai General Hospital, at a photon dose rate of 0.5 Gy/min. Dosimetry was performed with an ionization chamber and ferro-sulfate. X-ray high-intensity radiation was performed with a multi-RAD 225 irradiator (Faxitron, IL, USA) with 110 kV voltage, 10 mA current, 0.5 filters, 2 Gy for in vitro experiments, and 8 Gy and 6 Gy for in vivo experiments.

### 2.4. Plasmids and Cell Transfection

The pcDNA3.1-LATS2, vector plasmids, and siLATS2 plasmids were purchased from Genera Biotechnology (Shanghai, China). Cells were seeded in six-well plates and transfected with plasmids with Lipofectamine 3000 (Invitrogen, Carlsbad, CA, USA), following the manufacturer’s protocol.

### 2.5. RT-PCR and Illumina Hiseq xten/Nova seq 6000 Sequencing

RNA was isolated with TRIzol reagent (Invitrogen). cDNA was synthesized from 1 μg of total RNA according to the Takara protocol. The genes of interest were amplified using appropriate primers with 40 cycles. The primers sequences were as follows: LATS2, TGGGCTGGAGCAGAAGATGAC, GGGACAAAGGCGGAAAGGA. GAPDH, ATGATGACCCTTTTGGCTCC, CGTATTGGGCGCCTGGTCAC. The synthesized cDNA was subjected to end-repair, phosphorylation, and ‘A’ base addition according to Illumina’s library construction protocol. Libraries were size selected for cDNA target fragments of 300 bp on 2% Low-Range Ultra Agarose followed by PCR amplified using Phusion DNA polymerase (NEB) for 15 PCR cycles. After quantification by TBS380, paired-end RNA-seq sequencing library was sequenced with the Illumina HiSeq xten/NovaSeq 6000 sequencer (2 ×150 bp read length). Clean reads were separately aligned to reference genome with orientation mode using HISAT2 (http://ccb.jhu.edu/software/hisat2/index.shtml, accessed on 25 September 2019) software. The mapped reads of each sample were assembled by StringTie (https://ccb.jhu.edu/software/stringtie/index.shtml?t=example, accessed on 25 September 2019) in a reference-based approach. To identify differential expression genes (DEGs) between two different samples, the expression level of each transcript was calculated according to the transcripts per million reads (TPM) method. RSEM (http://deweylab.biostat.wisc.edu/rsem/, accessed on 25 September 2019) was used to quantify gene abundances. Essentially, differential expression analysis was performed using the DESeq2/DEGseq/EdgeR with *Q* value ≤ 0.05. DEGs with |log2FC| > 1 and *Q* value ≤ 0.05 (DESeq2 or EdgeR)/*Q* value ≤ 0.001 (DEGseq) were considered to be significantly different expressed genes. In addition, functional enrichment analysis, including GO and KEGG, were performed to identify which DEGs were significantly enriched at Bonferroni-corrected *p*-value ≤ 0.05, compared with the whole-transcriptome background. GO functional enrichment and KEGG pathway analyses were carried out by Goatools (https://github.com/tanghaibao/Goatools, accessed on 25 September 2019) and KOBAS (http://kobas.cbi.pku.edu.cn/home.do, accessed on 25 September 2019).

### 2.6. Clonogenic Assays

SiHa and C33A cells were treated with SFN (10 μM and 7.5 μM, respectively), transfected with LATS2 overexpression, or knocked down for 2 days, and then cultured in capsules at a density of 1000 cells per capsule after treatment. After around 2 weeks of incubation, colonies with more than 50 cells were produced and photographed.

### 2.7. Cell Immunofluorescence Staining

Cells were grown on coverslips and were fixed with 4% paraformaldehyde for 20 min, blocked with mixture of 5% bovine serum albumin and 0.2% Triton X-100 for 30 min, and incubated with antibodies against LATS2 (1:100, Proteintech, Wuhan, China), γ-H2AX (1:400, Cell Signaling Technology), Rad51 (1:300, Abcam), and MDC1 (1:100, Proteintech) overnight at 4 °C, followed by secondary antibodies CoraLite 594-conjugated goat anti-rabbit IgG (1:100, Proteintech) for 1 h at room temperature. Meanwhile, nuclei were counterstained with 4′6-diamidino-2-phenylindole (DAPI) for 5 min. The fluorescence images were captured with laser-scanning microscopy (Leica, Heidelberg, Germany).

### 2.8. Western Blot

Western Blot was performed according to standard protocols, as previously described. In brief, 30 µg protein samples were loaded into 11% sodium dodecyl sulfate-polyacrylamide (SDS) gel and then transferred onto polyvinylidene fluoride (PVDF) membranes (Millipore, Bedford, MA, USA) at 300 mA for 1.5 h. Membranes were blocked in 5% nonfat milk for 1.5 h and then incubated with specific primary antibodies overnight at 4 °C. Membranes were washed with phosphate-buffered saline (PBS) three times before incubation with appropriate secondary antibody for 1 h at room temperature. Lastly, the proteins were detected with an enhanced chemiluminescence kit (NcmECL Ultra, Shanghai, China). The original western blot image shown in Figures S1–S4.

### 2.9. In Vitro Proliferation Assays

Cells were cultured in 96-well culture plates at 3 × 10^3^ cells per well. CCK-8 reagent was added to wells with different treatments. The absorbance of each well was measured at different time points at 450 nm using by a microplate reader (Dojindo, Kumamoto, Japan). The percent growth inhibition or growth promotion at 24 h or 48 h was calculated by the following formula: 100 − [OD_SFN_/OD_control_] × 100, where OD_SFN_ and OD_control_ represented the absorbance at 450 nm.

### 2.10. Cell Apoptosis Analysis

SiHa and C33A cells were cultured and treated with 20 µM and 15 µM SFN, respectively, and were transfected with LATS2 overexpression or a control carrier. To analyze cell apoptosis, cells were collected and washed with ice-cold PBS 3 times 48 h after treatment. Then, cells were incubated with PE Annexin V and propidium iodide (PI) according to the PE Annexin V apoptosis detection kit I (BD Pharmingen, San Diego, CA, USA) protocol and were analyzed by BD FACSCalibur.

### 2.11. In Vivo Analysis

Female athymic nude mice at 6 weeks of age were purchased from the Shanghai General Hospital Experimental Animal Center of the Chinese Academy of Science. In xenograft tumor formation assays, mice were randomly separated into two groups (five per group). SiHa cells were subcutaneously injected into mice at a concentration of 1 × 10^7^ cells per mouse. One group of mice was injected intraperitoneally with SFN (25 μM), while the other group was set as control. They both were exposed to 8 Gy irradiation and the survival days were recorded. We reduced the radiation dose to 6 Gy and the mice were sacrificed 2 weeks after irradiation. We compared the tumor size between the SFN and the control groups. Mice were under euthanasia procedure of cervical dislocation. The experiment was carried out in strict accordance with the guide for care and use of laboratory animals and was approved by the department of laboratory animal science at Shanghai Jiaotong University School of Medicine.

### 2.12. Statistical Analysis

The difference between groups was analyzed by Student’s *t*-test with GraphPad Prism v 5.0 (GraphPad, San Diego, CA, USA). Each data point represented the mean of triplicates (*N* = 3) with the SEM indicated by error bars. Survival curves were examined by Kaplan–Meier analysis with the log-rank test analysis. *p*-value < 0.05 was considered statistically significant. * *p* < 0.05, ** *p* < 0.01, *** *p* < 0.001, **** *p*  < 0.0001.

## 3. Results

### 3.1. SFN Inhibits the Viability of Cervical Cancer Cells and Increases the Expression of LATS2

**Figure 1:**
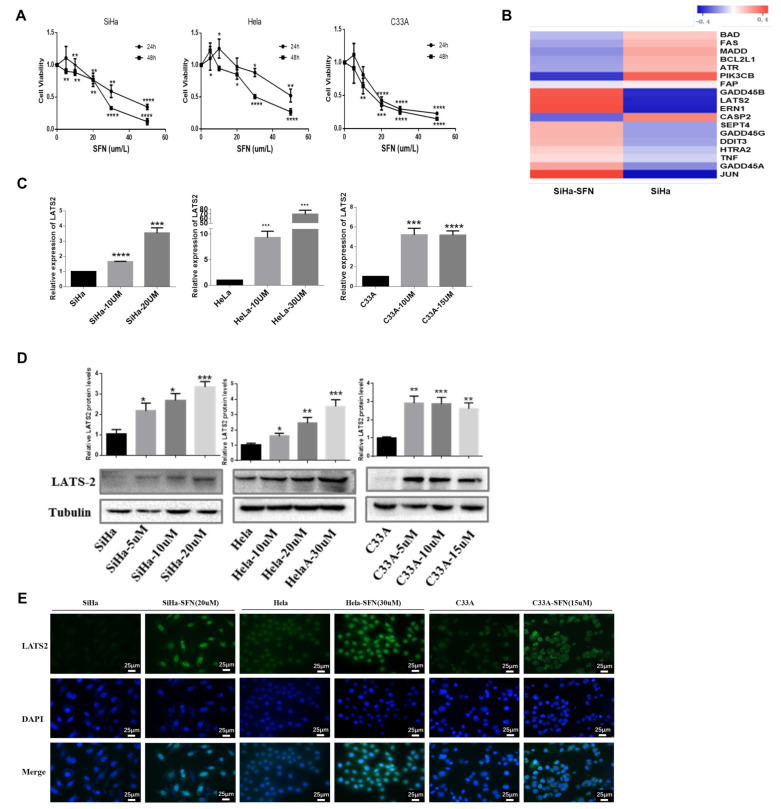
SFN inhibits cell proliferation and promotes LATS2 expression. (**A**) SiHa, HeLa, and C33A cells were treated with accelerating concentrations of SFN, from 5 μM to 50 μM. Cell viability was evaluated by CCK8 assay at 24 h and 48 h. (**B**) RNA-Seq analysis of SiHa cells comparing genes expression involving cycle arrest and apoptotic functions between SFN treatment (25 μM) and the blank. (**C**,**D**) LATS2 mRNA and protein level were investigated after 48 h of SFN treatment with RT-PCR and Western blotting. Tubulin was used as a loading control. Cropped blots were showed in Western blotting. The samples derived from the same experiment and those blots were processed in parallel. (**E**) LATS2 was examined by the immunofluorescence in SiHa, C33A, and HeLa cells after treatment of SFN for 48 h. * *p* < 0.05, ** *p* < 0.01, *** *p* < 0.001, **** *p*  < 0.0001.

To explore the antitumor activity of SFN in cervical cancer cells, we examined the effects of SFN treatment on the proliferation of cervical cancer cells. SiHa, HeLa, and C33A cell lines were treated with SFN, with concentrations ranging from 5 μM, 10 μM, 20 μM, 30 μM, to 50 μM. Cell viability was analyzed by the CCK8 method at different time points (24 h, 48 h). We found that SFN inhibited the growth of cervical cancer cells in a time- and dose-dependent manner (Figure 1A). Concentrations of 25 μM and 15 μM are most close to IC50 of SFN in SiHa cells and C33A cells, respectively. To search for the targets of SFN, we conducted mRNA sequencing (Table S1) to identify differentially expressed mRNAs in SiHa cells treated with SFN (25 uM, 48 h), from which we picked the most differentially expressed genes involving apoptosis and cell cycle arrest function (Table S2), and then a heatmap was undertaken (Figure 1B). SFN-treated SiHa cells showed accelerated expression of several mRNA including JUN, ERN1, LATS2, and GADD45B. The ERN1-JNK-JUN pathway and the complex nature of c-Jun regulation functions on oncogenic networks have been investigated after two decades of extensive efforts [15,16,17,18]. One study has illustrated HPV E6 oncogene activated JNK1/2 phosphorylation and demonstrated a positive feedback loop between JNK-dependent activation of the EGFR pathway and HPV E6/E7 expression in cervical cancer [19]. LATS2 mRNA was also significantly upregulated when SFN treated, and no study has illustrated the role of LATS2 in cervical cancer. Thus, we further verified the impact of SFN treatment on LATS2 expression in cervical cancer cells. We performed qRT-PCR analyses and the results showed that SFN upregulated the mRNA levels of LATS2 (Figure 1C). As shown in Figure 1D, protein expression level of LATS2 was increased when SFN was treated. Immunoblot analysis showed a marked increase in LATS2 immune fluorescence in cell lines treated with SFN (Figure 1E).

### 3.2. Overexpression of LATS2 Suppresses Cervical Cancer Cell Tumorigenicity In Vitro

**Figure 2:**
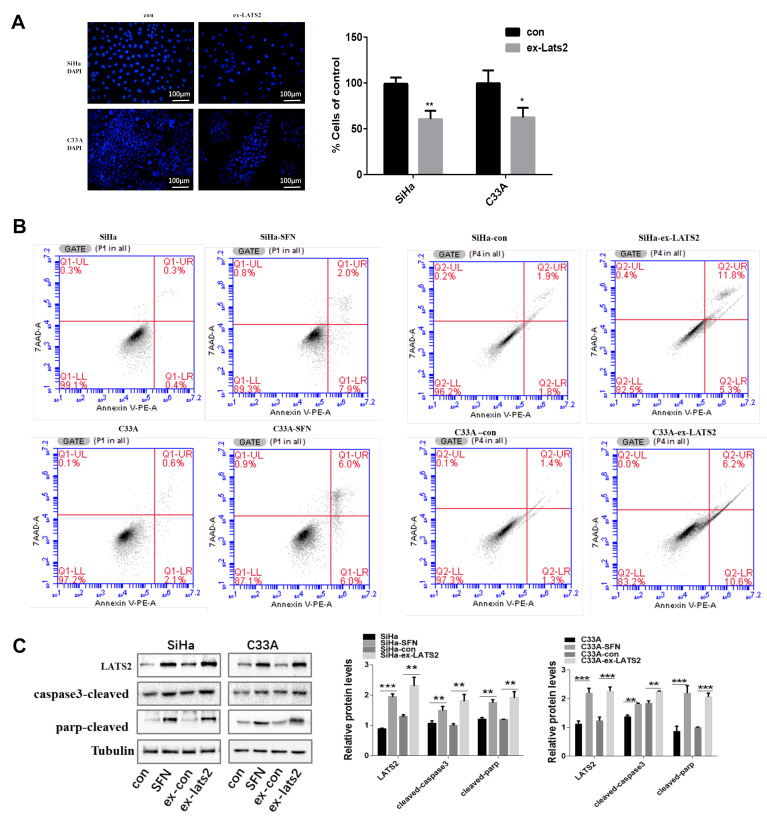
LATS2 suppresses cervical cancer cell proliferation and promotes apoptosis. (**A**) Proliferation of SiHa and C33A with LATS2-overexpressing cells and their respective controls was assessed by DAPI staining and nuclear counting using CellProfiler. (**B**) Cell apoptosis was evaluated by flow cytometric analysis. SiHa and C33A were treated with SFN (20 and 15 μM, respectively) for 48 h, or transfected with LATS2 overexpression plasmid (ex-LATS2) or control vector. The data shown are from a single representative experiment performed in triplicate. (**C**) Western blot analysis of the levels of cleaved caspase-3 and cleaved PARP. Cropped blots were showed in Western blotting. The samples derived from the same experiment and those blots were processed in parallel. * *p* < 0.05, ** *p* < 0.01, *** *p* < 0.001.

To investigate whether LATS2 alters the biological characteristics of cervical cancer cells, we attempted to overexpress LATS2 in the HPV (+) SiHa cells line and the HPV (−) C33A cells line by transfecting cells with plasmids PcDNA3.1-LATS2. The overexpression of LATS2 in these cells was validated by Western blotting in Figure 2C. As shown in Figure 2A, we found that increased LATS2 expression significantly inhibited cell proliferation. Next, cell apoptosis was analyzed by Annexin V-PE and 7AAD staining. In SiHa and C33A cells, SFN treatment and LATS2 overexpression both promoted cell apoptosis compared with the control group, particularly the process of early apoptosis (Figure 2B). To further validate the proapoptotic role of LATS2, we examined two biochemical markers of apoptosis, cleaved PARP and cleaved Caspase-3, the expressions of which were enhanced in SFN treatment group and LATS2 overexpression group compared with the control group, respectively (Figure 2C).

### 3.3. SFN Enhances Radiosensitivity In Vitro by Inhibiting DNA Damage Repair

**Figure 3:**
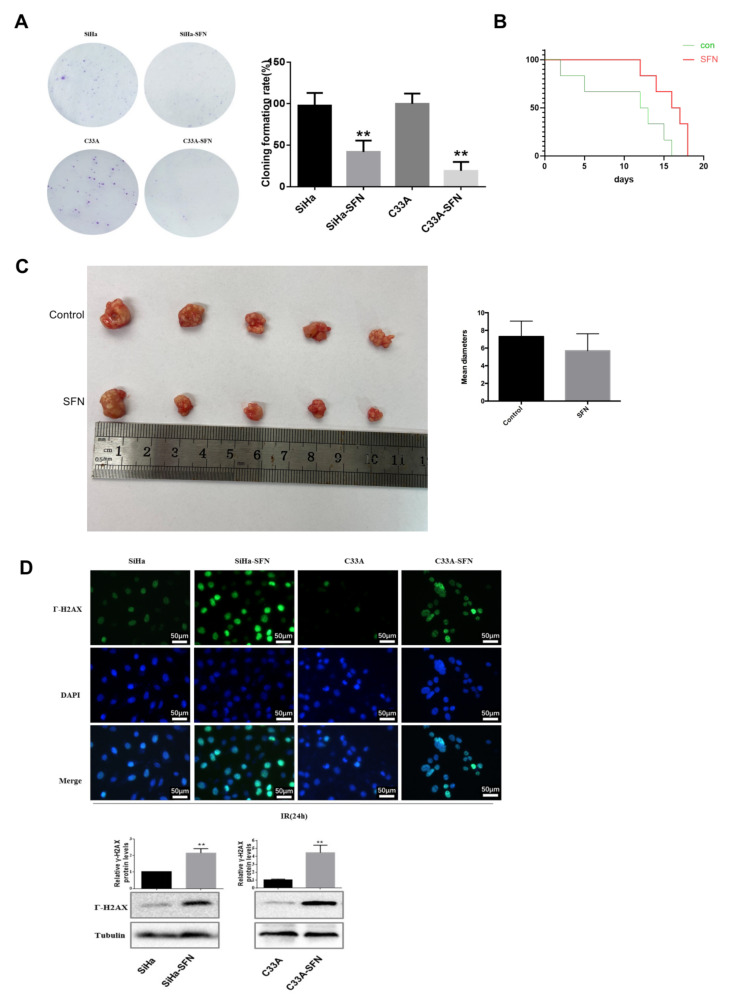

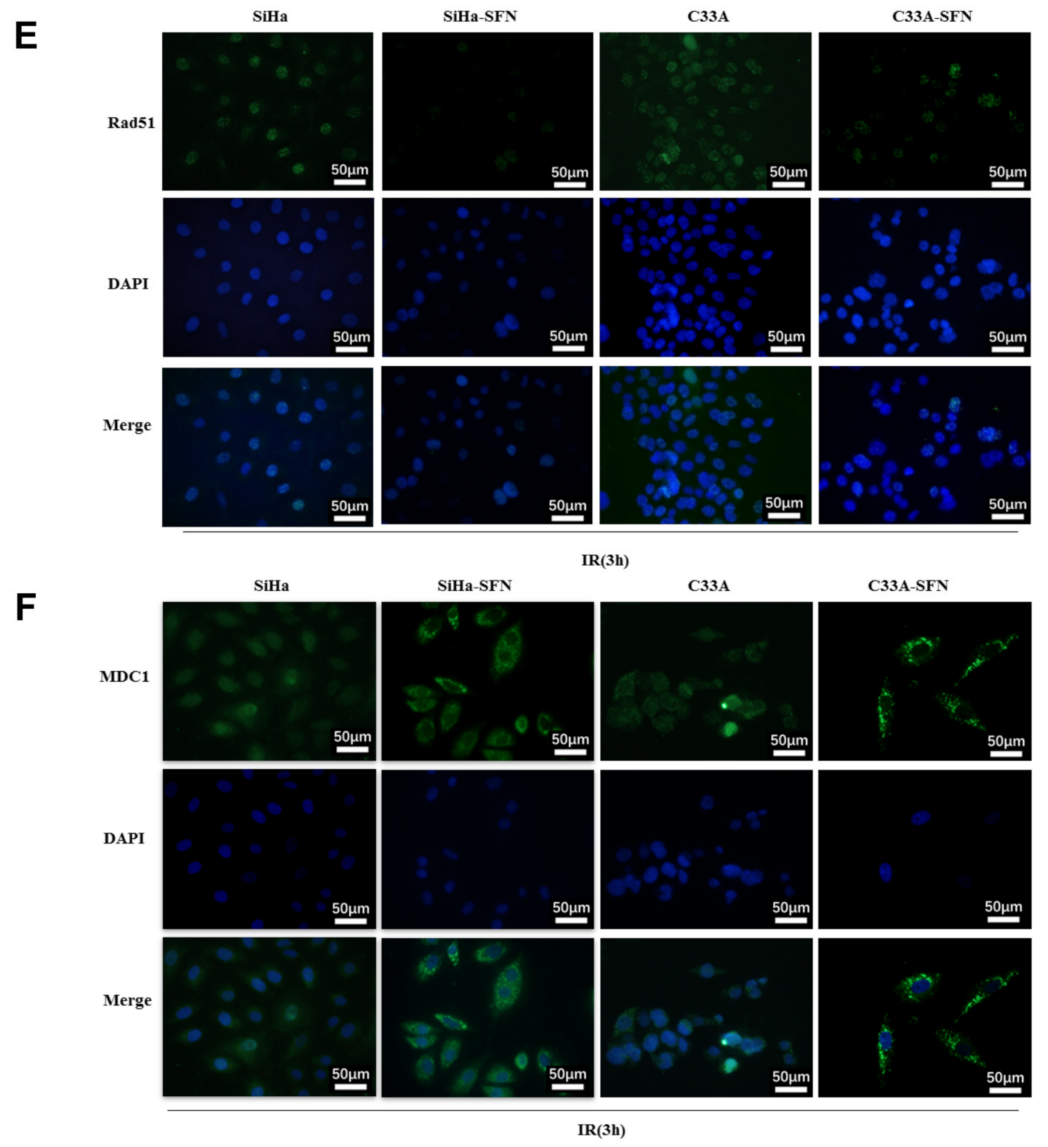
SFN enhances radiosensitivity in vitro by inhibiting RAD51 and MDC1 nuclear recruitment. (**A**) Colony formation of SiHa and C33A cells treated with SFN (10 and 7.5 uM, respectively) or control after irradiation. After 2 weeks of incubation, colonies with more than 50 cells were produced. (**B**) Kaplan–Meier plot of survival days of mice with SFN treatment (25 uM) and control group after IR exposure (8 Gy) (**C**) Tumors of mice injected with SiHa cells were removed 2 weeks after injection. (**D**) Immunofluorescence analysis of DNA damage according to the presence of γ-H2AX in SiHa and C33A cells treated with SFN (15 and 10 uM, respectively), 24 h after irradiation. Western blotting was conducted to quantify γ-H2AX protein levels 24 h after being subjected to IR. Cropped blots were showed in Western blotting. The samples derived from the same experiment and those blots were processed in parallel. (**E**,**F**) Representative immunofluorescence images of Rad51 and MDC1 nucleus foci formation 3 h after irradiation comparing cells with SFN treatment and control. ** *p* < 0.01.

To examine the impact of SFN treatment on radiation sensitivity of cervical cancer cells, we used colony formation analysis to compare cell survivals after irradiation exposure. SiHa and C33A were exposed to 2 Gy irradiation after SFN treatment (10 μM and 7.5 μM, respectively). We found that SFN treatment significantly restrained colony formation compared with the control group after radiotherapy (Figure 3A). In vivo, the median survival days of mice with SFN treatment (25 μM) and IR exposure (8 Gy) was 16.5 days, which was statistically longer than that of control group with IR exposure (12.5 days) (Figure 3B). We reduced the radiation dose to 6 Gy and found the SFN group ended up with smaller size of tumor than the control group 2 weeks after IR, but the difference was not statistically significant (Figure 3C).

Since DNA damage is one of the major contributing factors for the loss of clonogenic survival upon absorbed radiation, DSBs are particularly injurious to cells and the incompetent repair can lead to cell death. We first quantified the accumulation of γH2AX, a DNA damage marker [20], as shown in Figure 3D. γH2AX was higher-expressed in nucleus in SFN-treated cells compared with control cells 24 h after irradiation. Western blot showed that the protein level of γ-H2AX after irradiation in the SFN treatment group was higher than that in control group, suggesting the reduced repairment of the IR-induced DNA damage after SFN treatment. So, we suspected that disrupted DNA damage response upon SFN treatment in combination with IR would result in unrepaired DSBs and thereby maintained high levels of DSBs.

DSBs in the mammalian genome were repaired through homologous recombination (HR) and non-homologous end-joining (NHEJ) repair pathways. Rad51 is a crucial component of the HR pathway. Rad51, MDC1 forms foci that colocalized extensively with γ-H2AX and promoted recruitment of repair proteins to the site of DNA breaks within minutes after irradiation. We tested the RAD51 immunofluorescence in cells postradiotherapy, and found that RAD51 foci was significantly decreased in the nucleus 3 h after irradiation in SFN-treated cells compared with control cells (Figure 3E). Additionally, immunofluorescence analysis detected that the foci of MDC1 accumulated was decreased in nucleus and increased in the cytoplasm 3 h after irradiation in SFN-treated cells than in control cells (Figure 3F). These results suggested that SFN treatment suppressed RAD51 and MDC1 nucleus recruitment and DNA damage repair, especially HR, after irradiation, thereby leading to a slower damage removal and inhibited survival of cervical cancer cells.

### 3.4. LATS2 Has Made a Contribution Role in SFN-Facilitated Reduced DNA Damage Repair

**Figure 4:**
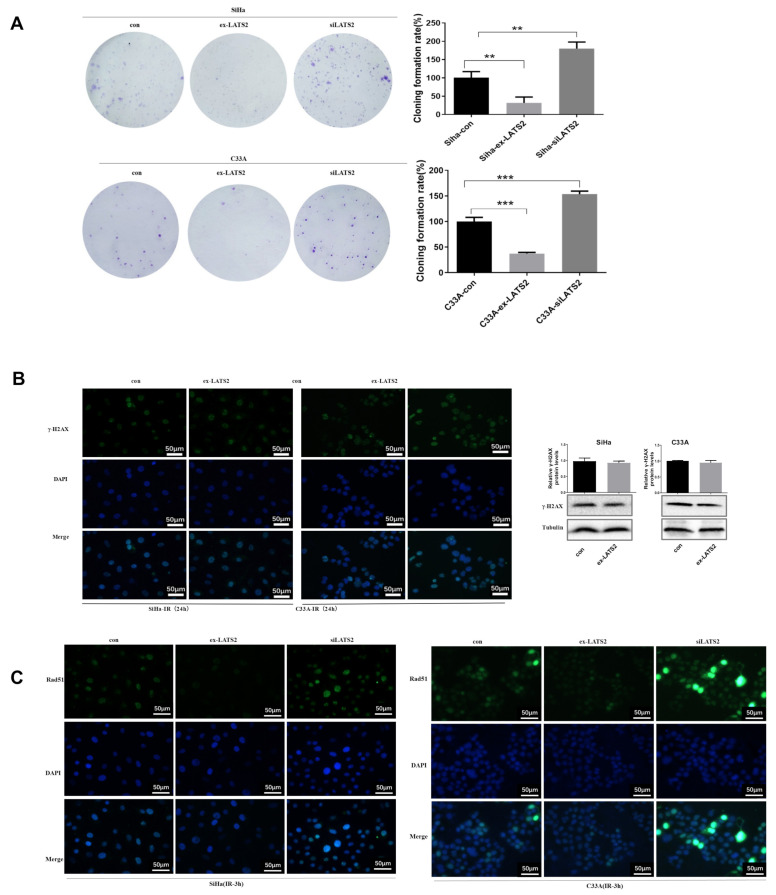

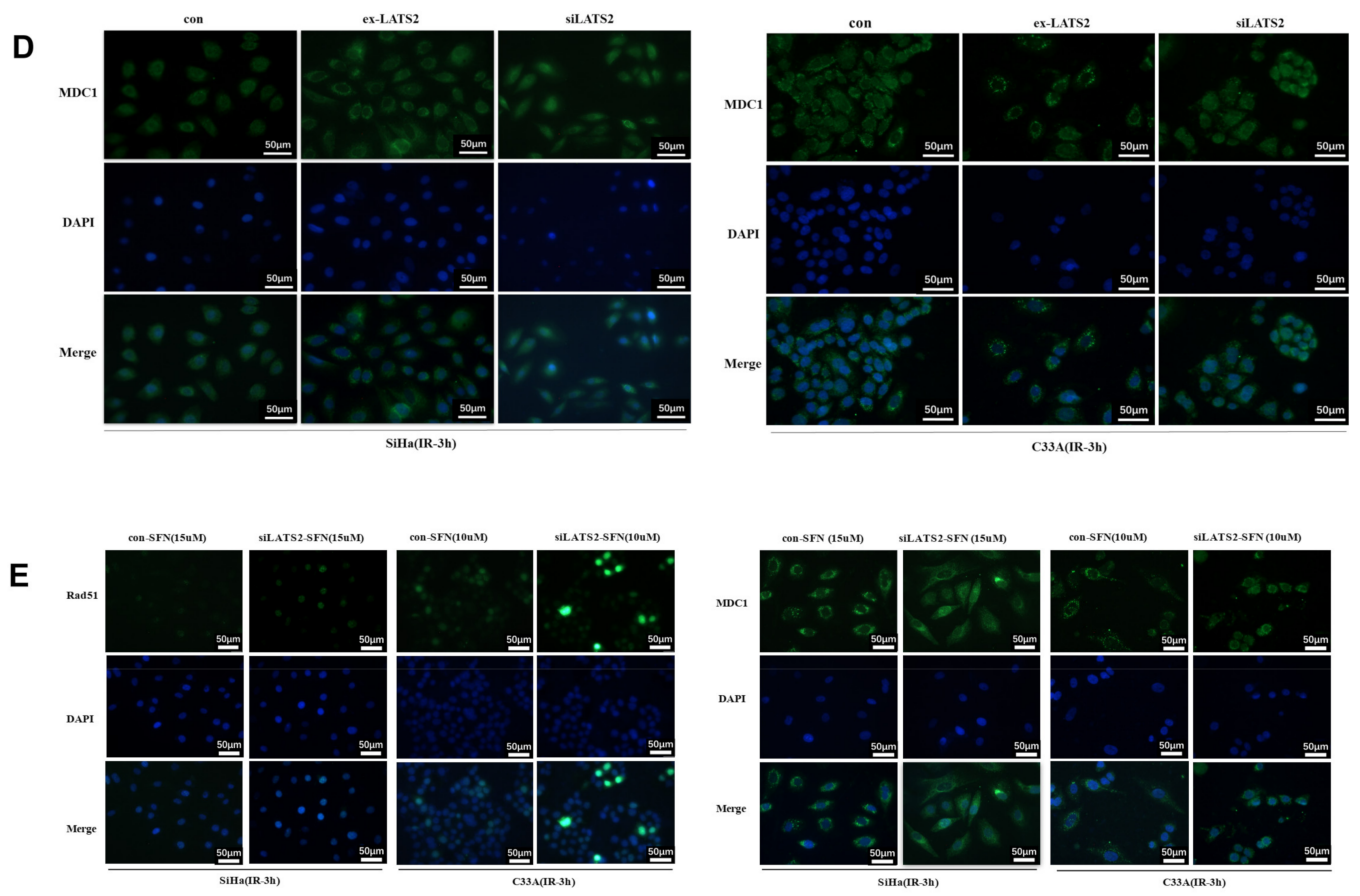
Effect of LATS2 on DSB response. (**A**) Colony formation after irradiation in SiHa and C33A cells with LATS2 overexpression and LATS2 knockdown. Colonies of >50 cells were produced after 2 weeks of incubation. (**B**) Immunofluorescence analysis of DNA damage according to the presence of γH2AX in SiHa and C33A cells with LATS2 overexpression or control, and Western blotting was conducted to quantify γ-H2AX protein levels 3 h after being subjected to IR. Cropped blots were shown in Western blotting. The samples derived from the same experiment and those blots were processed in parallel. (**C**,**D**) Representative immunofluorescence images of Rad51 and MDC1 nucleus foci formation 3 h after irradiation in cells with LATS2 overexpression and LATS2 knockdown. (**E**) Representative immunofluorescence image of Rad51 and MDC1 nucleus foci formation 3 h after irradiation in SiHa and C33A cells of LATS2 knockdown and SFN treatment, compared with SFN treatment. ** *p* < 0.01, *** *p* < 0.001.

We tried to estimate the role of LATS2 in DNA damage repair process. Firstly, we discovered that LATS2 overexpression suppressed the cervical cancer cells survival after irradiation and LATS2 knockdown enhanced the colony formation compared with the control group (Figure 4A). It is noteworthy that LATS2 overexpression did not induce significant change of γ-H2AX expression in 24 h after irradiation, as detected by immunofluorescence and Western blotting (Figure 4B). However, Rad51 nuclear accumulation was inhibited by LATS2 overexpression and enhanced by LATS2 knockdown 3 h after irradiation compared with the control group (Figure 4C). Meanwhile, less MDC1 accumulation in the nuclei and more in the cytoplasm was found in LATS2-overexpressing cells 3 h after exposure to radiation. There was more foci formation of MDC1 in the nucleus of LATS2 knockdown cells compared with the control group (Figure 4D). This indicated that MDC1 transferred from nucleus to cytoplasm after irradiation in the LATS2-overexpressing group, reminiscent of the effect when SFN was treated. These results suggested that LATS2 overexpression inhibited DNA damage repair especially HR in cervical cancer cells after irradiation.

In order to investigate the role of LATS2 in this SFN-inhibited DNA damage repair process and Rad51, MDC1 nucleus aggregation, we compared the irradiated SFN-treated LATS2-knockdown cells and irradiated SFN-treated control cells. Additionally, we found the accumulation of Rad51 and MDC1 in the nucleus was higher in LATS2-knockdown group than the control group (Figure 4E), which indicated LATS2 loss intervened with SFN-alleviated RAD51 and MDC1 nucleus accumulation and resumed the repair of DNA damage. Taken together, these observations suggested SFN treatment resulted in inhibition of HR pathway thereby making the cancer cells relatively sensitive to radiotherapy. LATS2 overexpression has made an indispensable contribution to this SFN-facilitated radiotherapy sensitization.

## 4. Discussion

In this study, we identified SFN as radiotherapy sensitizer of cervical cancer cells and LATS2 as a novel downstream target gene of SFN. We found that the mRNA and protein levels of LATS2 were accelerated in SFN-treated cells. SFN treatment and LATS2 overexpression can both inhibit cell proliferation and promote apoptosis by upregulating cleaved caspase-3 and cleaved PARP. SFN treatment and LATS2 overexpression also inhibited cervical cancer survival after irradiation by suppressing Rad51 and MDC1 nucleus recruitment and DNA damage repair. SFN treatment resulted in inhibition of HR pathway and LATS2 has made an indispensable contribution to this SFN-facilitated radiotherapy sensitization.

Cervical cancer with high-risk or intermediate-risk factors from pathological examination after radical hysterectomy should be supplemented with postoperative adjuvant radiotherapy [21]. Irradiation therapy is the main treatment of advanced cervical cancer. Ionizing radiation (IR) kills proliferating cancer cells by causing replicating dependent DNA double strand breaks (DSBs), which is the main type of DNA damage. IR-induced DNA damage repair mainly occurs during the S phase [22]. Many reports have shown that an increase in the proportion of cells in G2/M phase or a reduction in S phase after radiation was beneficial to enhance the radiosensitivity of cells [23,24,25]. Sulforaphane, a dietary phytochemical, acts as an epigenetic modulator and results in cell cycle arrest and cellular senescence in cervical cancer and breast cancer cell lines [26,27]. SFN has been harnessed targeting the vulnerability for radiation resistance of cervical cancer. Reports found that DNA fragmentation was induced by SFN and that DSB repair was inhibited in colon cancer cells and HeLa cells [28,29,30]. We found similar results in SiHa and C33A cells in our studies. The colony formation assay combined with 2 Gy irradiation revealed that the ability of colony formation in cells treated with SFN or LATS2 overexpression was significantly lower than that in the control cells. Additionally, mice with SFN treatment and irradiation survived longer than those without SFN treatment. These results confirmed SFN radiosensitization role in cervical cancer cells.

Large tumor suppressors (LATS2) are the homologues of drosophila warts and are serine/threonine kinases and core components in the hippo pathway [31,32]. In cervical cancer, HPV E6 and E7 oncoproteins maintained the inhibited hippo pathway and high Yap protein level, which regulated cervical cancer progression [33,34]. Activated LATS2 was able to promote the phosphorylation of the oncoproteins Yap and Taz, leading to cytoplasmic retention and degradation. Ma et al. found that LATS2 acts as a classic tumor suppressor in several kinds of cancer, including esophageal squamous cell carcinoma, breast cancer, pancreatic cancer, and endometrial cancer [35,36,37,38]. Activated LATS2 negatively regulates G1/S transition and led to the subsequent inhibition of cell proliferation and tumorigenesis, as well as the induction of apoptosis [39,40,41,42]. Additionally, LATS2 negatively regulates centrosome duplication and coordinates mitotic fidelity and genomic stability [43,44]. Conversely, loss of LATS2-induced dysregulation of hippo signaling might lead to organ overgrowth and tumors. However, the role of LATS2 in tumor suppression has not been fully elucidated in cervical cancer. We firstly revealed that LATS2 as a tumor suppressor in cervical cancer and LATS2 overexpression promoted tumor cells apoptosis. Meanwhile, LATS2 acted as a downstream modulator of SFN. SFN treatment enhanced IR sensitivity and accelerated LATS2 expression. The mechanism of SFN treatment modulation of LATS2 expression remain unexplored.

Once irradiation was performed and DNA damage occurred, ATM was activated and phosphorylated the tail of H2AX at Ser139 (γH2AX) on the chromatin flanking the DSB, which attracted combination of the mediator of DNA damage checkpoint protein 1 (MDC1). MDC1 took part in DNA damage sensing and signaling and constituted both a complex and a feedback loop resulting in amplification and stabilization of γH2AX. This served as a platform for recruitment and accumulation of other DNA repair factors, such as Rad51, BRCA1, BRCA2, and PALB2. RAD51 recombinase facilitated high-fidelity DNA damage repair by locating in the homology in sister chromatids and enabled DNA strand invasion into the sister chromatid [45,46]. Rad51 nuclear foci and the nuclear accumulation of MDC1—to some extent—represented the DNA damage repair, especially the HR pathway [47]. According to our data, the quantity and intensity of Rad51 foci and MDC1 in the nucleus of SFN-treated and LATS2-overexpressing cells were obviously decreased compared with those in the control cells, indicating that SFN treatment and LATS2 overexpression inhibited the DNA damage repair, especially the HR pathway. We found that the recruitment of MDC1 in the nucleus was blocked upon LATS2 overexpression and SFN treatment, resulting in the impaired formation of Rad51 foci under ionizing radiation in cervical cancer cells. These results are in accordance with research by Zhang in MCF-7 cells that MDC1 exerted an impact in Rad51-mediated homologous recombination by retaining Rad51 in chromatin [48]. Additionally, LATS2 knockdown attenuated the alleviation of Rad51 and MDC1 nucleus accumulation induced by SFN, and then, to some extent, resumed the repair of DNA damage, indicating that LATS2 served as one of the downstream targets of SFN which inhibited Rad51 and MDC1 taking part in DSB repair and enhanced the radiosensitivity of cervical cancer cells.

## 5. Conclusions

Our study provided the first evidence that SFN and LATS2 overexpression enhanced the radiosensitivity of human cervical cancer cells in vitro. In addition, we found that SFN and LATS2 overexpression inhibited RAD51 and MDC1 nucleus recruitment after radiation, partially contributing to suppressing DSB repair and enhancing the ability to undergo cells apoptosis. More broadly, results presented here illustrated that SFN acted as an approach to sensitize cervical cancer cells to irradiation. SFN served as the agonist of LATS2 and promoted cervical cancer radiosensitivity, and LATS2 might act as a radiotherapy-sensitive marker in the future. Prospectively, SFN could be added to platinum-based radiosensitizing chemotherapy between sessions of radiotherapy for advanced-staged cervical cancer patients in RCTs. Specifically, it is critical to explore the radiotherapy-sensitive marker role of LATS2 prospectively, and compare the SFN treatment effect and long-term overall survival.

## Data Availability

The data presented in this study are available on request from the corresponding author.

## Supplementary Materials

The following supporting information can be downloaded at: https://www.mdpi.com/article/10.3390/cancers14081872/s1, Figure S1. The original western blot for Figure 1D; Figure S2. The original western blots for Figure 2C; Figure S3. The original western blots for Figure 3D; Figure S4. The original western blots for Figure 4B; Table S1: mRNA sequence difference between SFN treatment and the blank; Table S2: The most differentially expressed mRNAs involving apoptosis and cell cycle arrest function.

## Abbreviations

SFN	sulforaphane
HR	homologous recombination
IR	ionizing radiation
DSB	DNA double-strand breaks
DDR	DNA damage repair
CCRT	concurrent chemoradiotherapy
NHEJ	non-homologous end joining
LATS2	large tumor suppressors 2
MDC1	DNA damage checkpoint protein 1
