# Supplementary material for: SFN Enhanced the Radiosensitivity of Cervical Cancer Cells via Activating LATS2 and Blocking Rad51/MDC1 Recruitment to DNA Damage Site
Source: Cancers (Basel). 2022 Apr 8;14(8):1872. doi: 10.3390/cancers14081872 (PMC9026704; doi:10.3390/cancers14081872)
Supplement: Supplementary file 1 [file cancers-14-01872-s001.zip › cancers-1553902-Figures S1-S4.pdf]

Supplementary Material: SFN Enhanced the Radiosensitivity of Cervical Cancer Cells via Activating LATS2 and Blocking Rad51/MDC1 Recruitment to DNA Damage Site

Shiyu Wang, Yanan Wang, Xiangnan Liu, Yongbin Yang, Sufang Wu and Yuan Liu

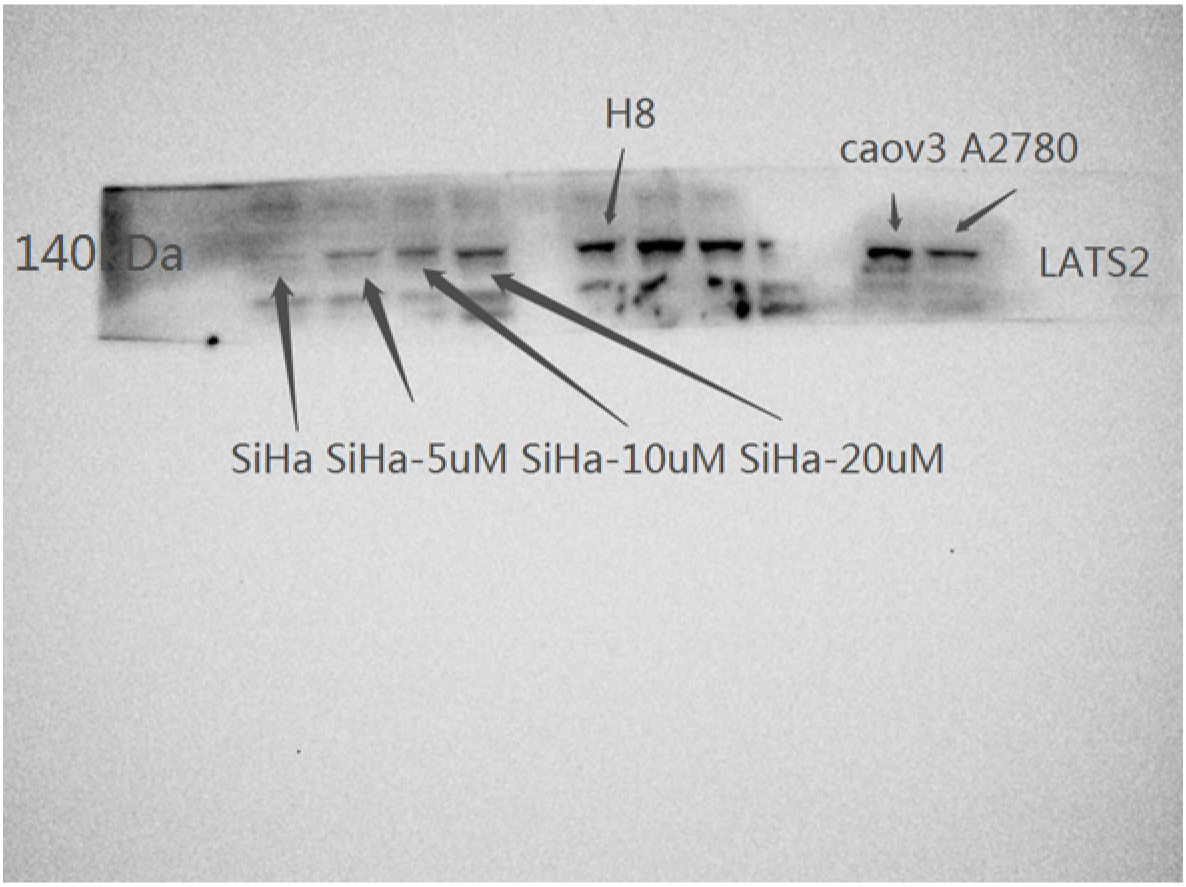

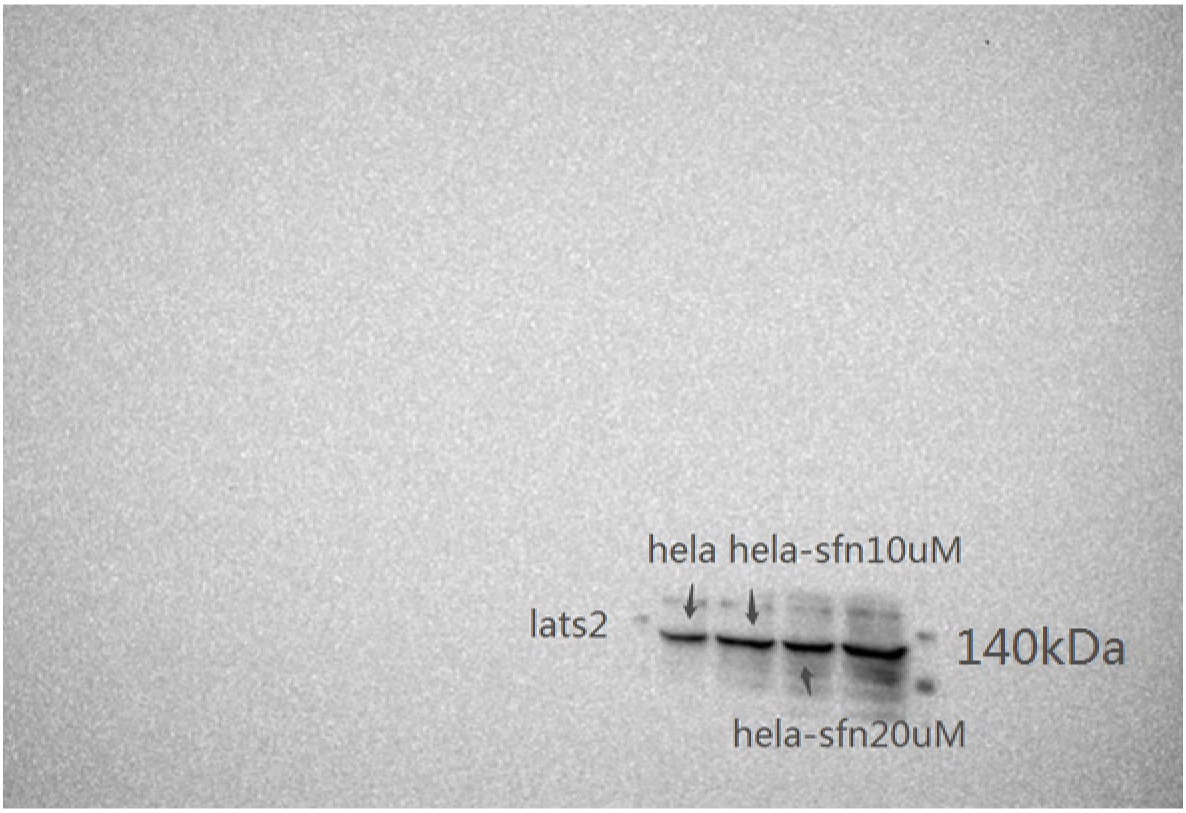
hela hela-sfn10uM
lats2
140kDa
hela-sfn20uM

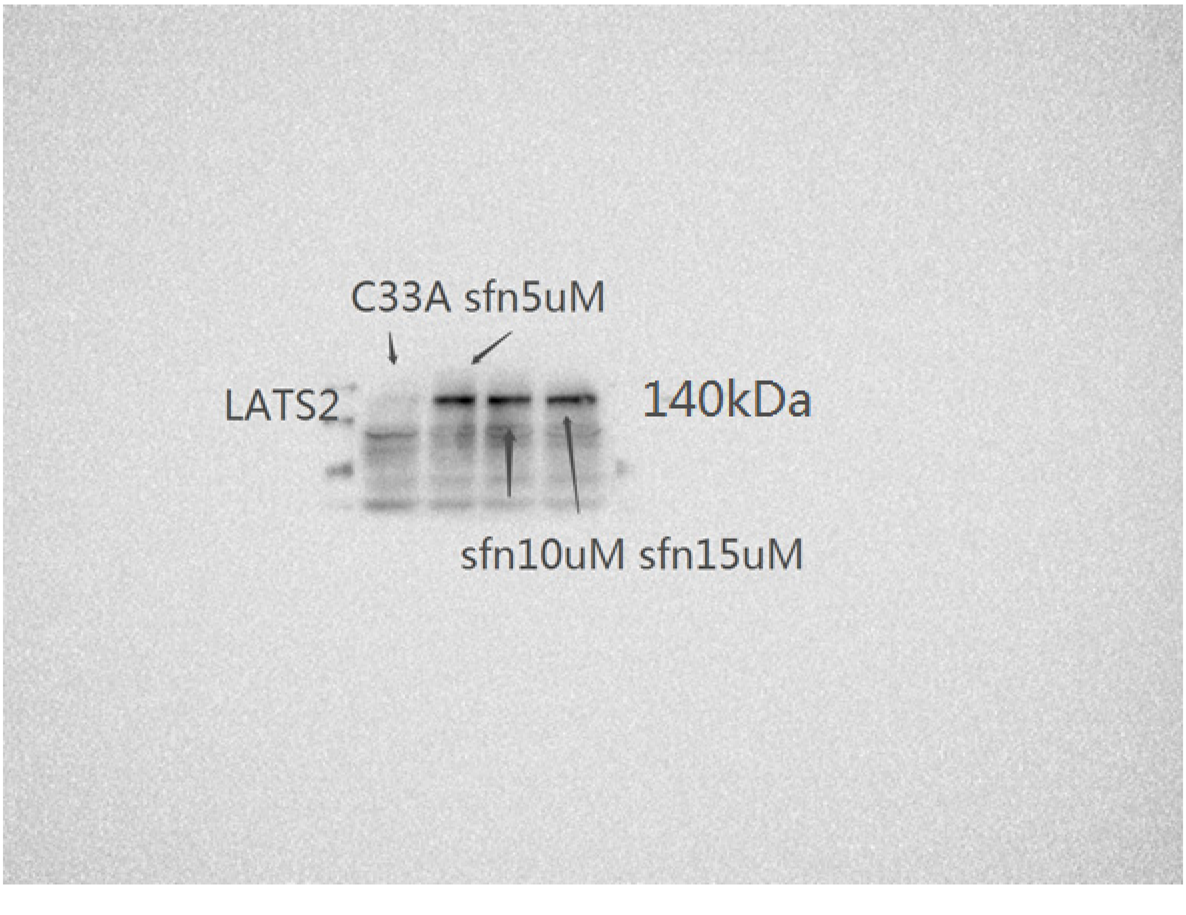
C33A sfn5uM
LATS2
140kDa
sfn10uM sfn15uM

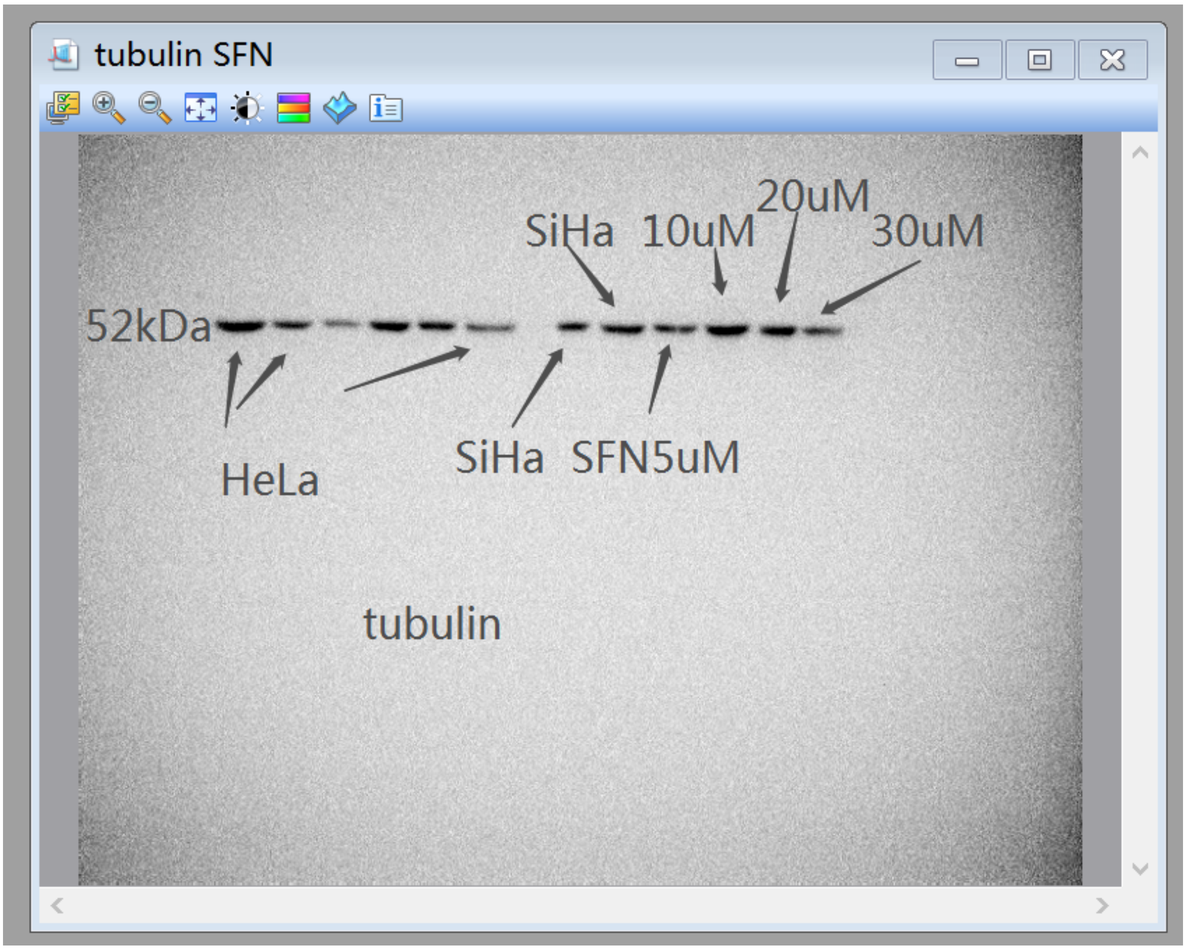
tubulin SFN
SiHa
10uM
20uM
30uM
52kDa
HeLa
SiHa
SFN5uM
tubulin

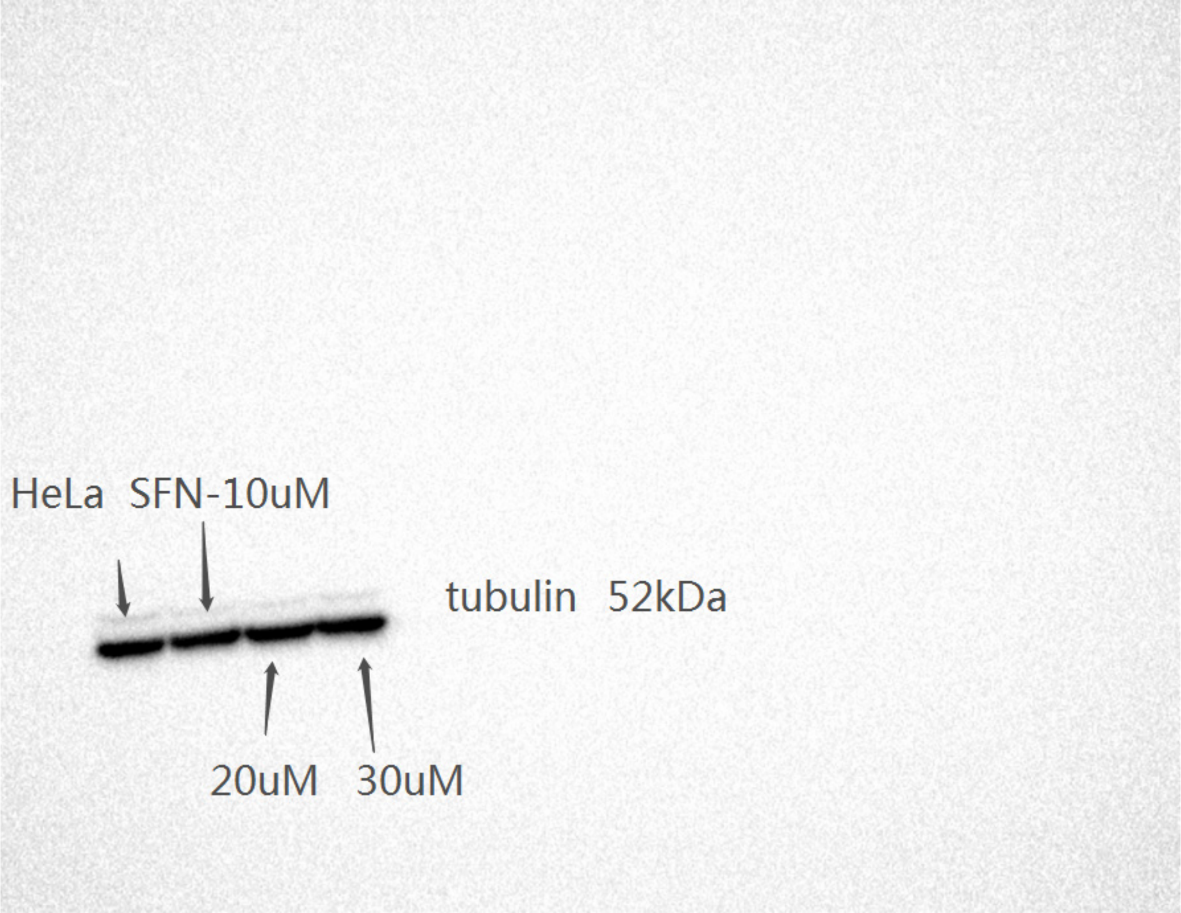

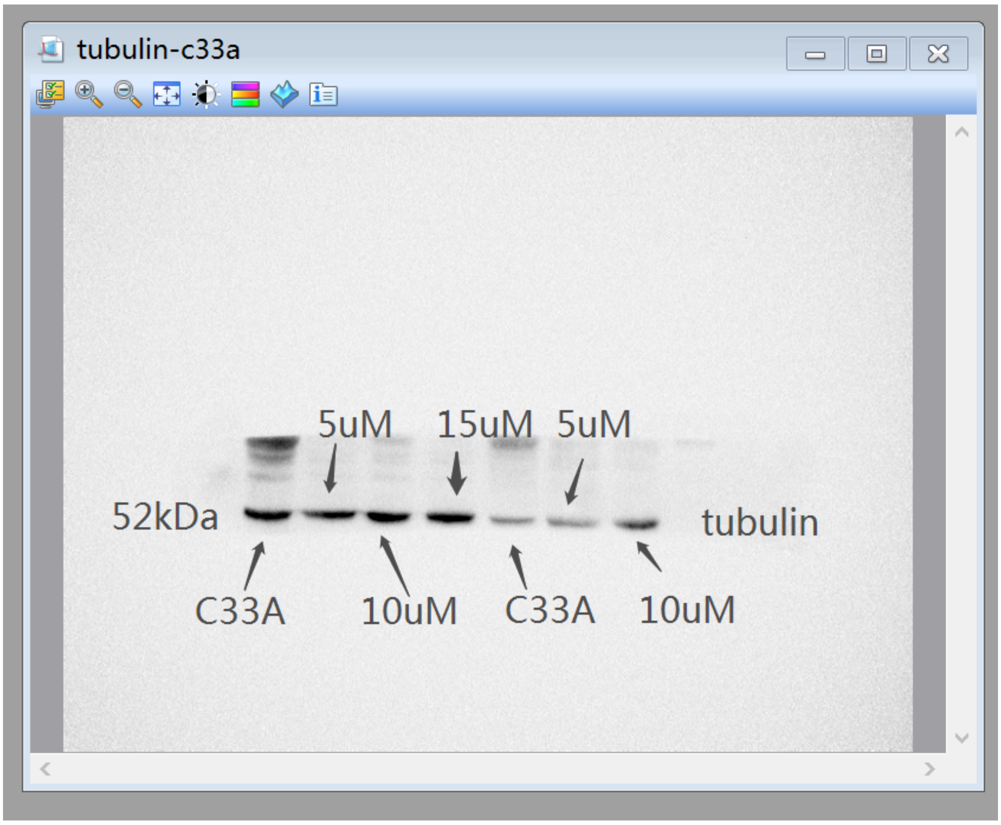

Figure S1. The original western blot for figures 1D.

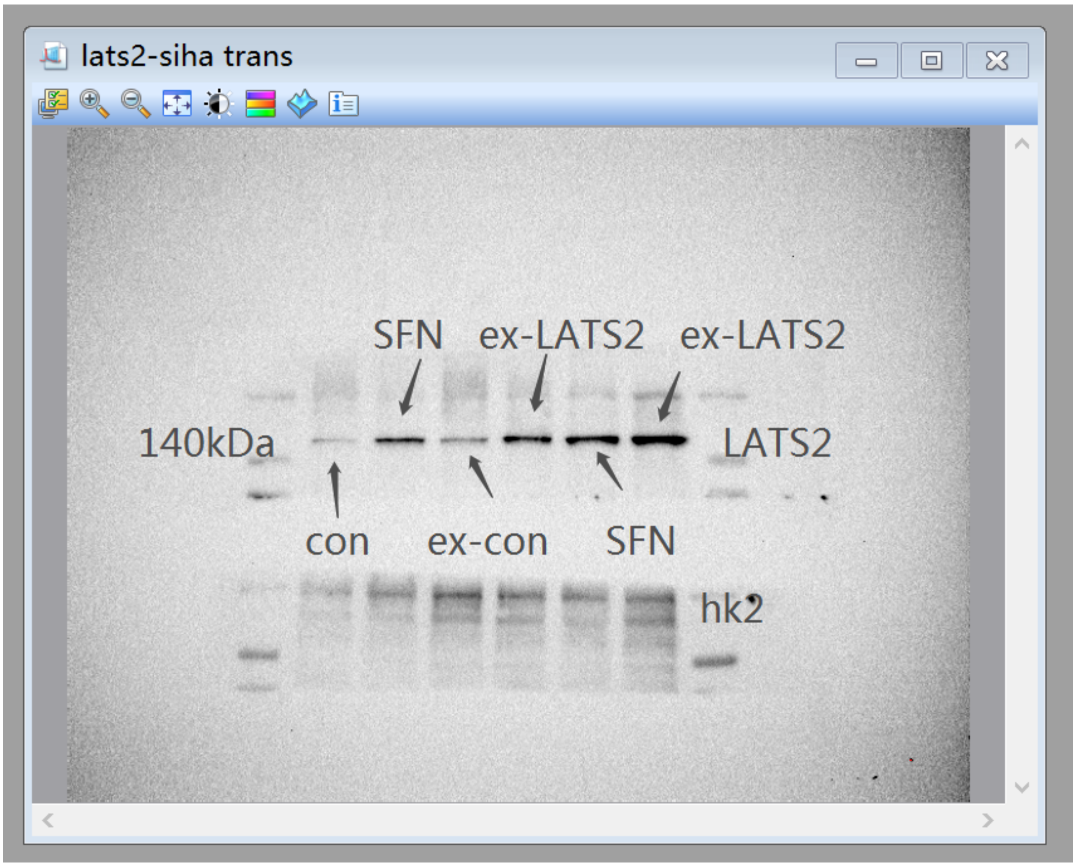
lats2-siha trans
SFN
ex-LATS2
ex-LATS2
140kDa
LATS2
con
ex-con
SFN
hk2

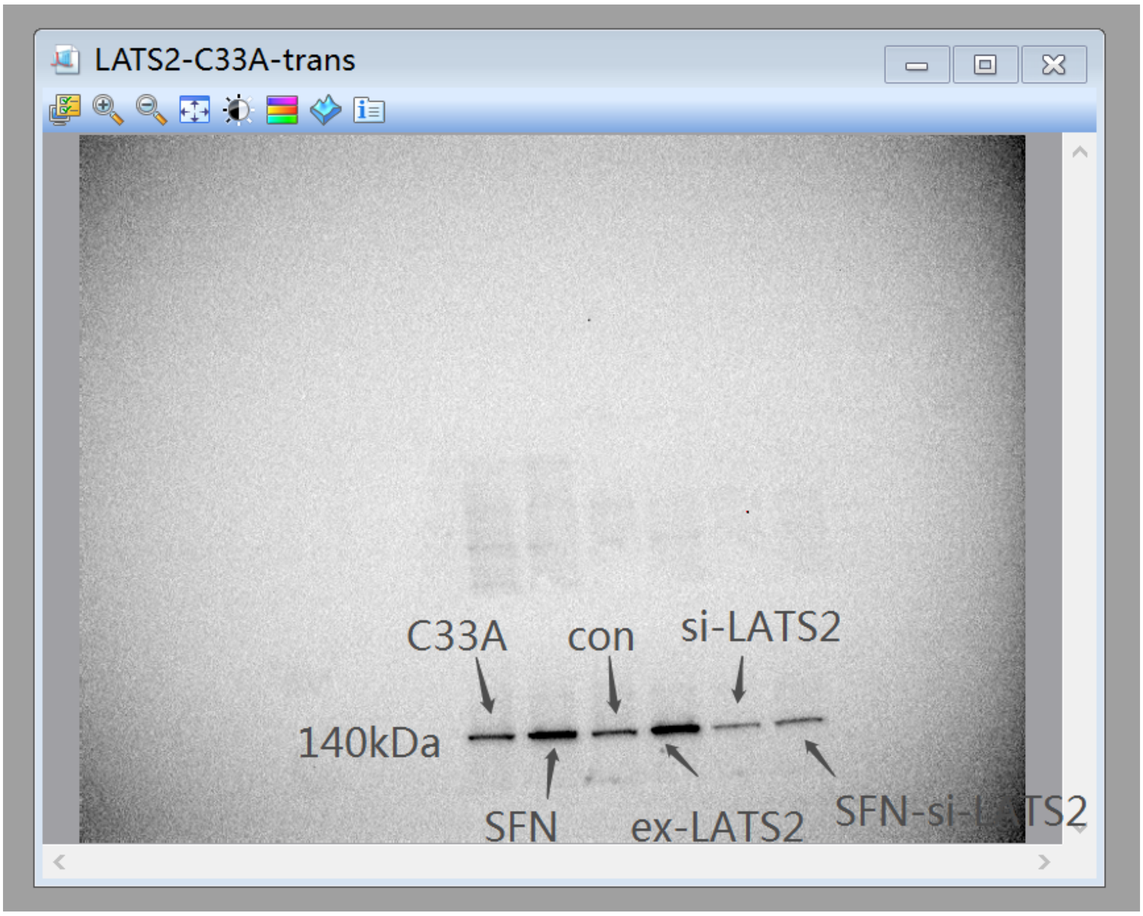
LATS2-C33A-trans
C33A
con
si-LATS2
140kDa
SFN
ex-LATS2

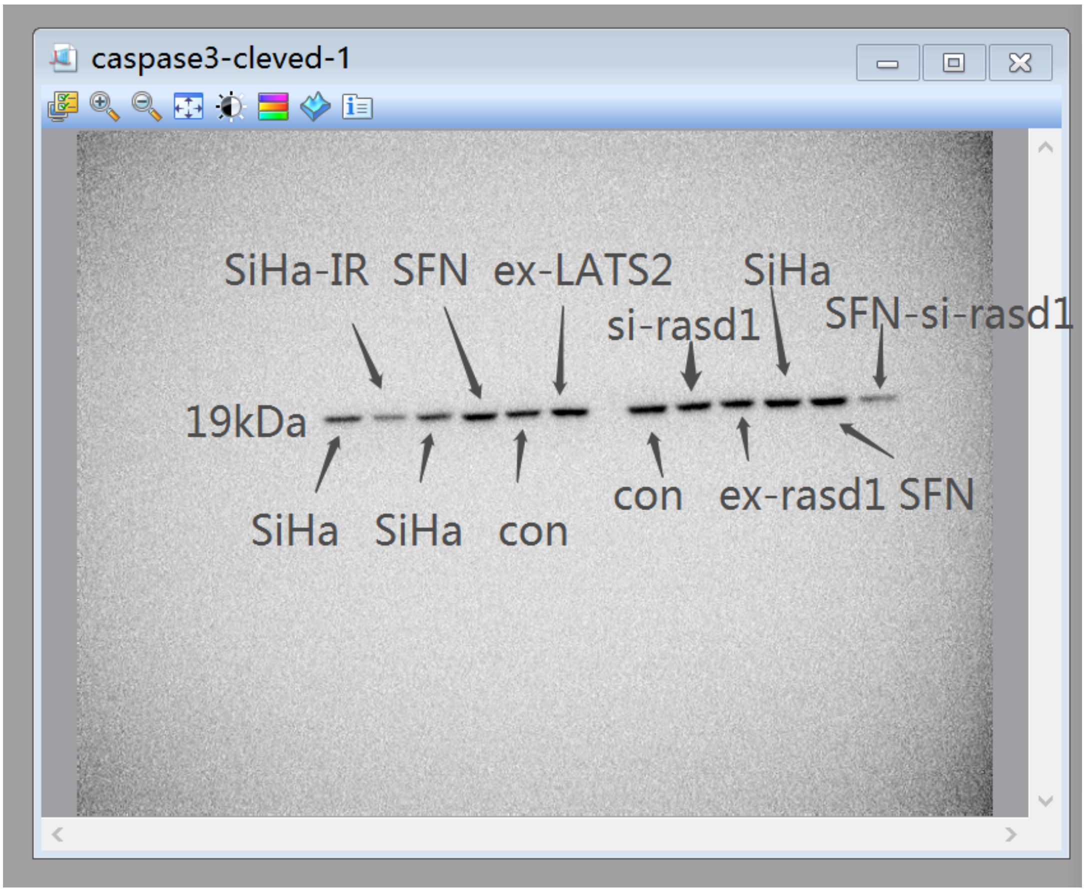
caspase3-cleved-1
SiHa-IR SFN ex-LATS2
SiHa
si-rasd1
SFN-si-rasd1
19kDa
SiHa SiHa con
con ex-rasd1 SFN

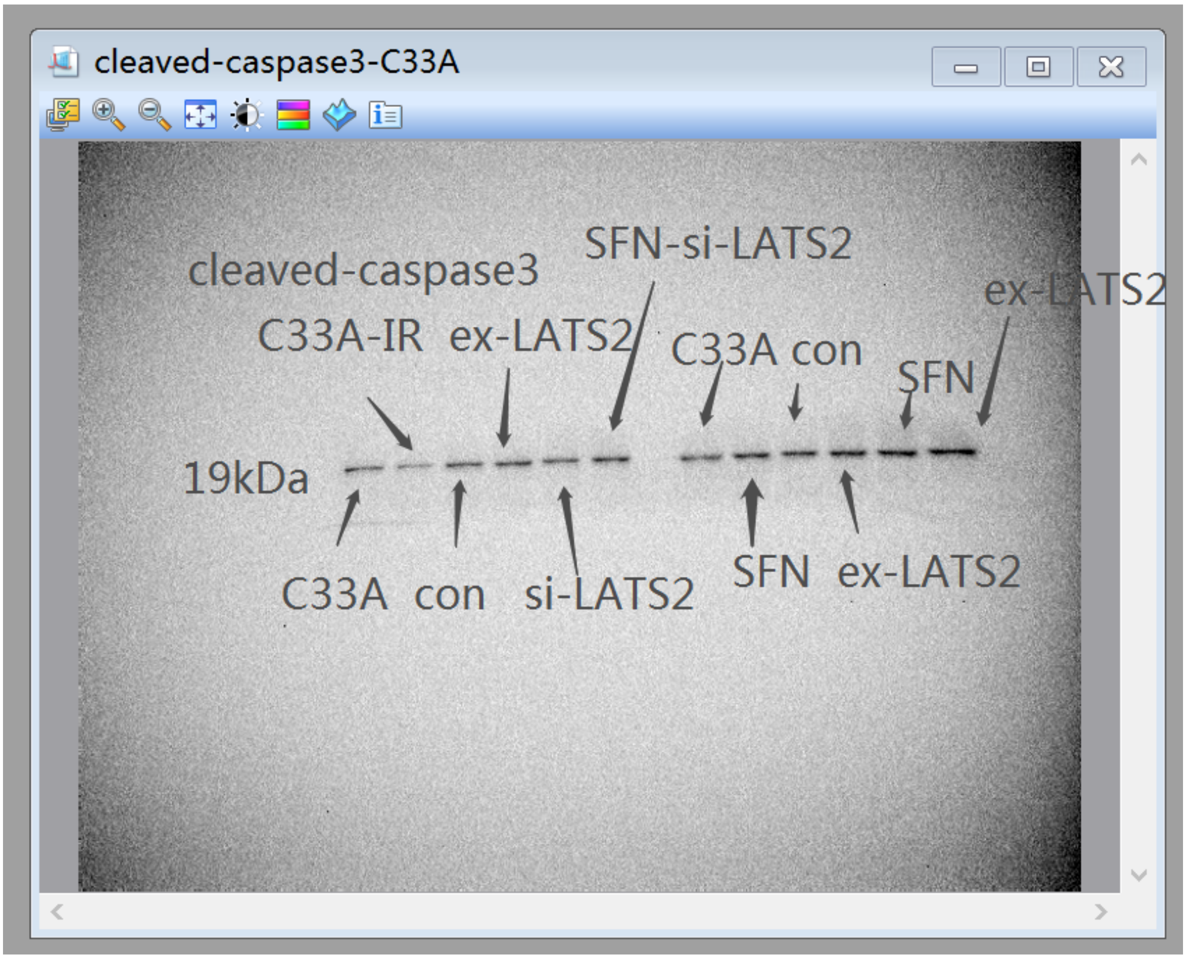
cleaved-caspase3-C33A
cleaved-caspase3
SFN-si-LATS2
ex-LATS2
C33A-IR
ex-LATS2
C33A con
SFN
19kDa
C33A
con
si-LATS2
SFN
ex-LATS2

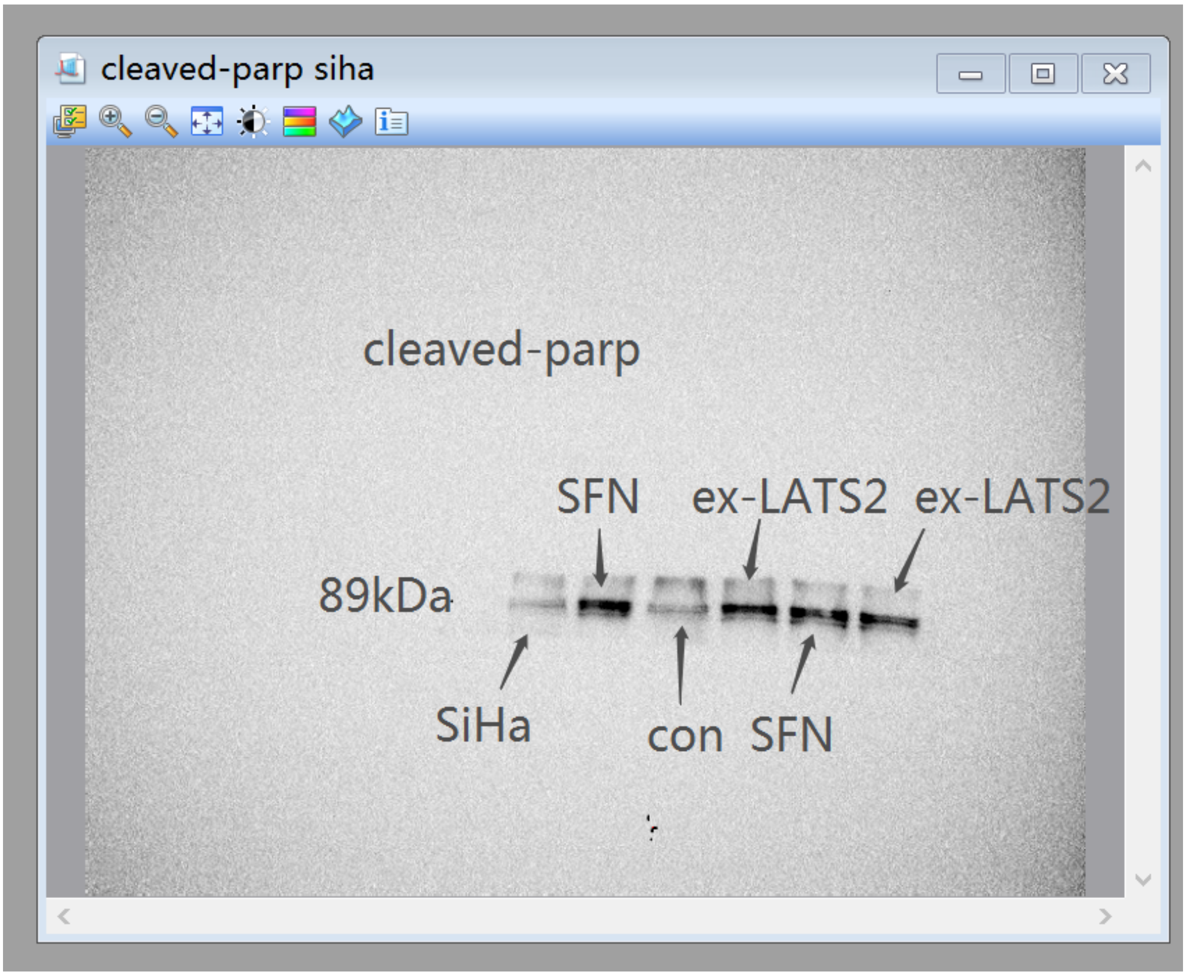
cleaved-parp siha
cleaved-parp
SFN
ex-LATS2
ex-LATS2
89kDa
SiHa
con
SFN

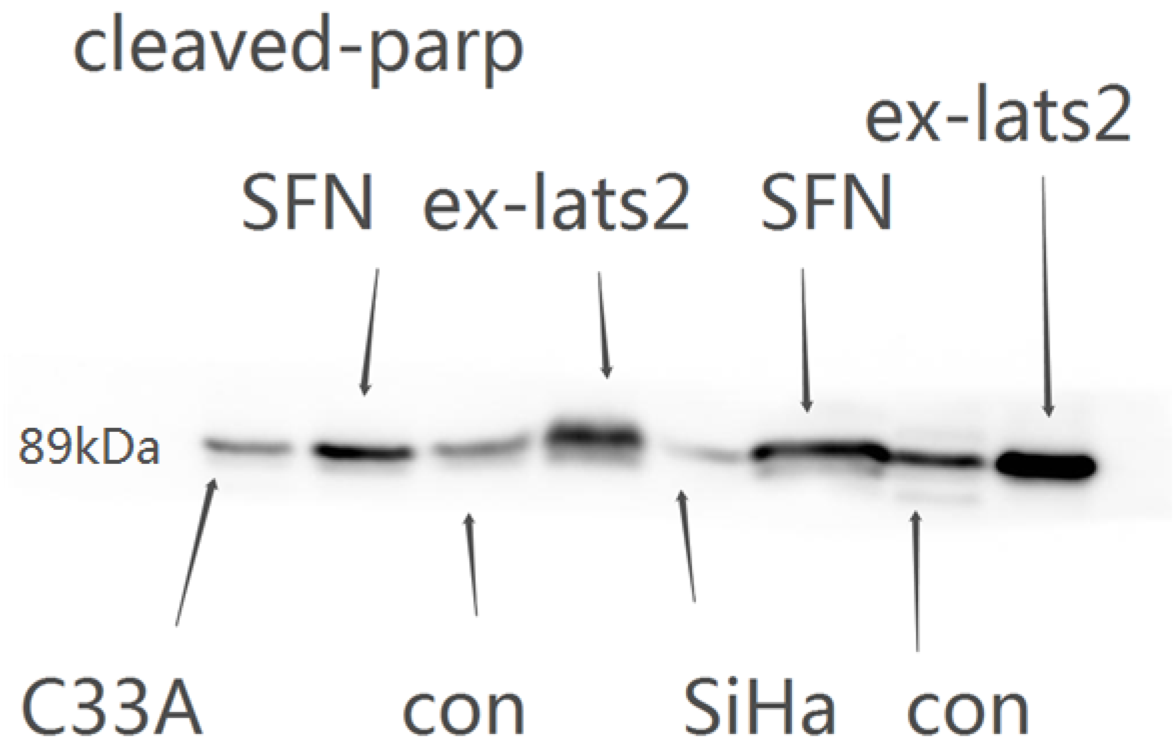
cleaved-parp
ex-lats2
SFN
ex-lats2
SFN
89kDa
C33A
con
SiHa
con

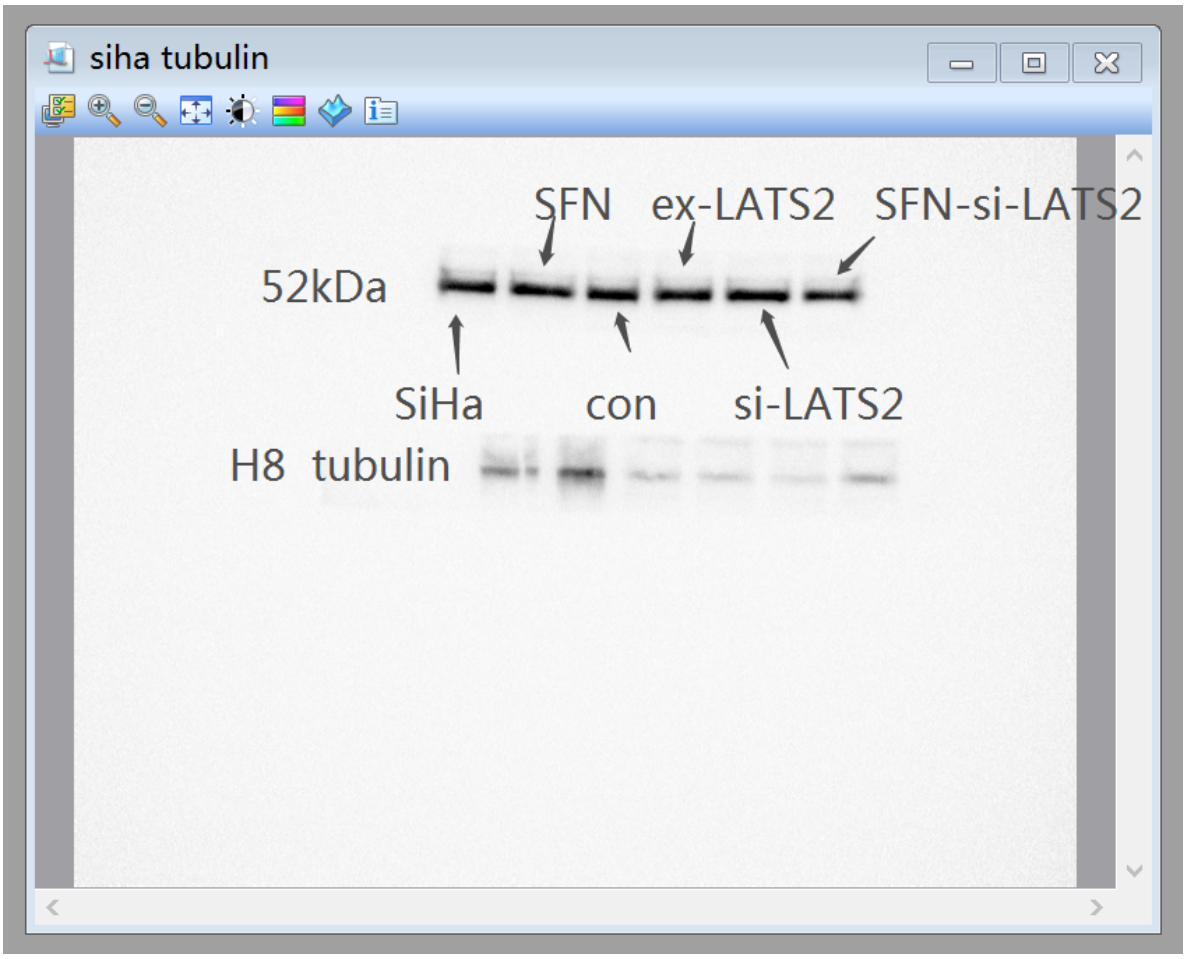
siha tubulin
SFN
ex-LATS2
SFN-si-LATS2
52kDa
SiHa
con
si-LATS2
H8 tubulin

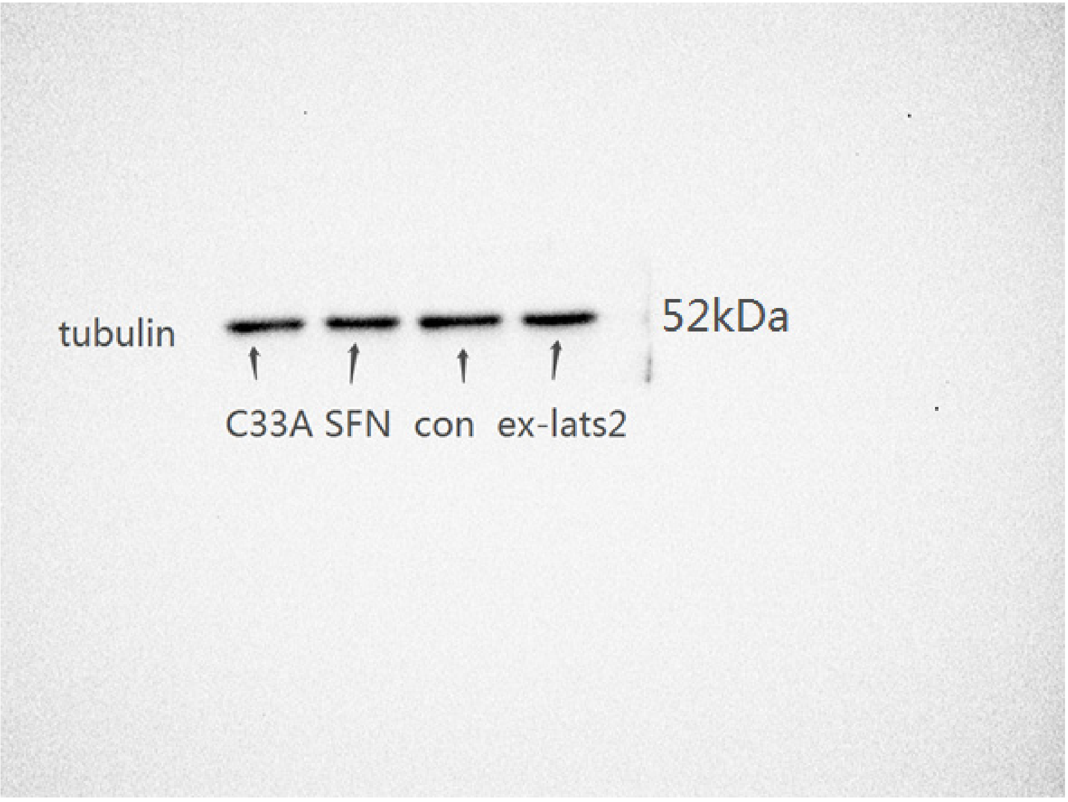

Figure S2. The original western blots for figure 2C.

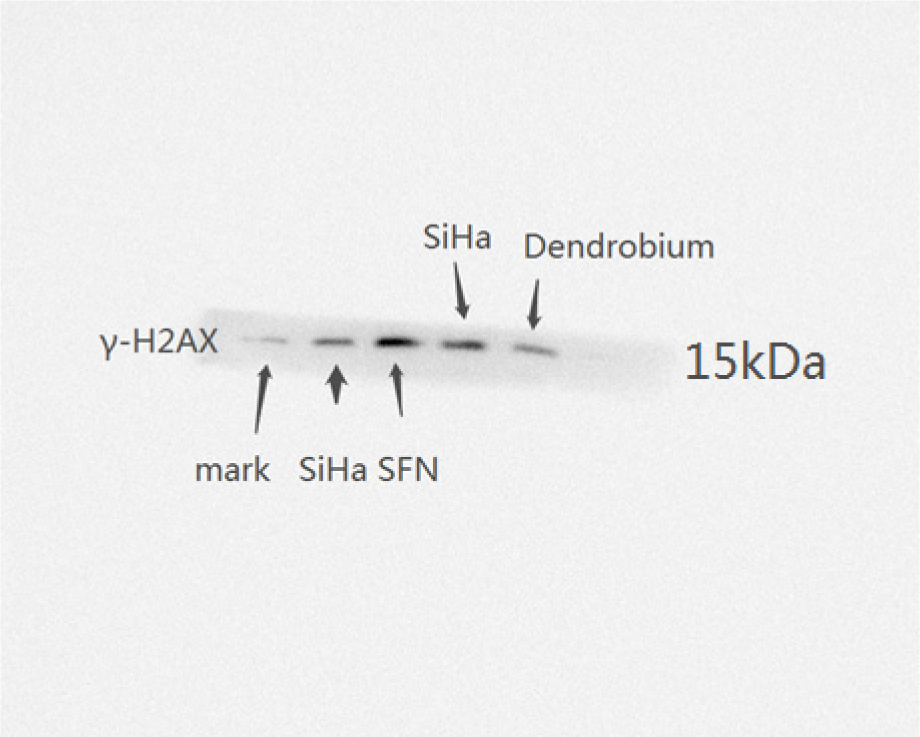

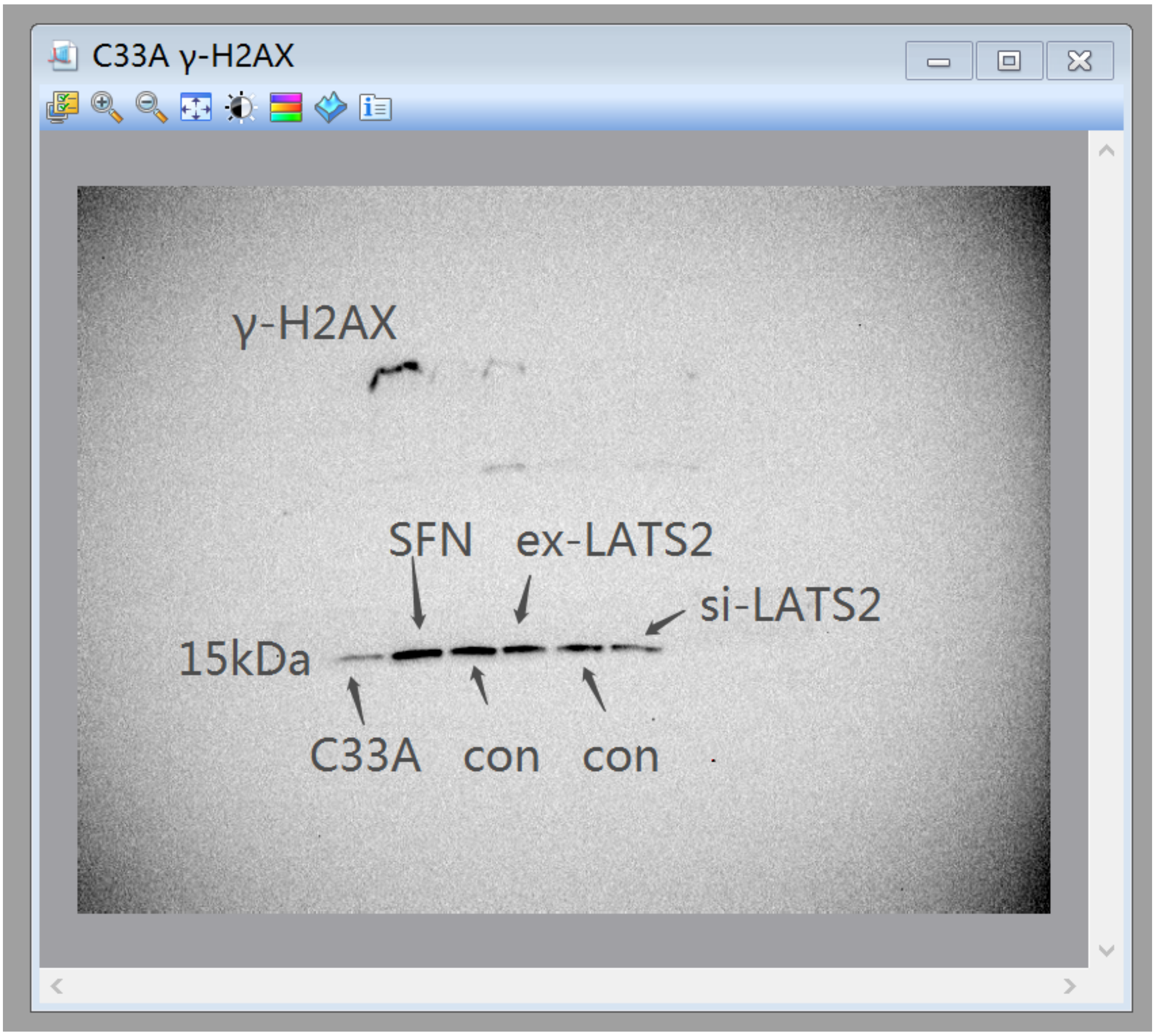
C33A γ-H2AX
γ-H2AX
SFN
ex-LATS2
si-LATS2
15kDa
C33A
con
con

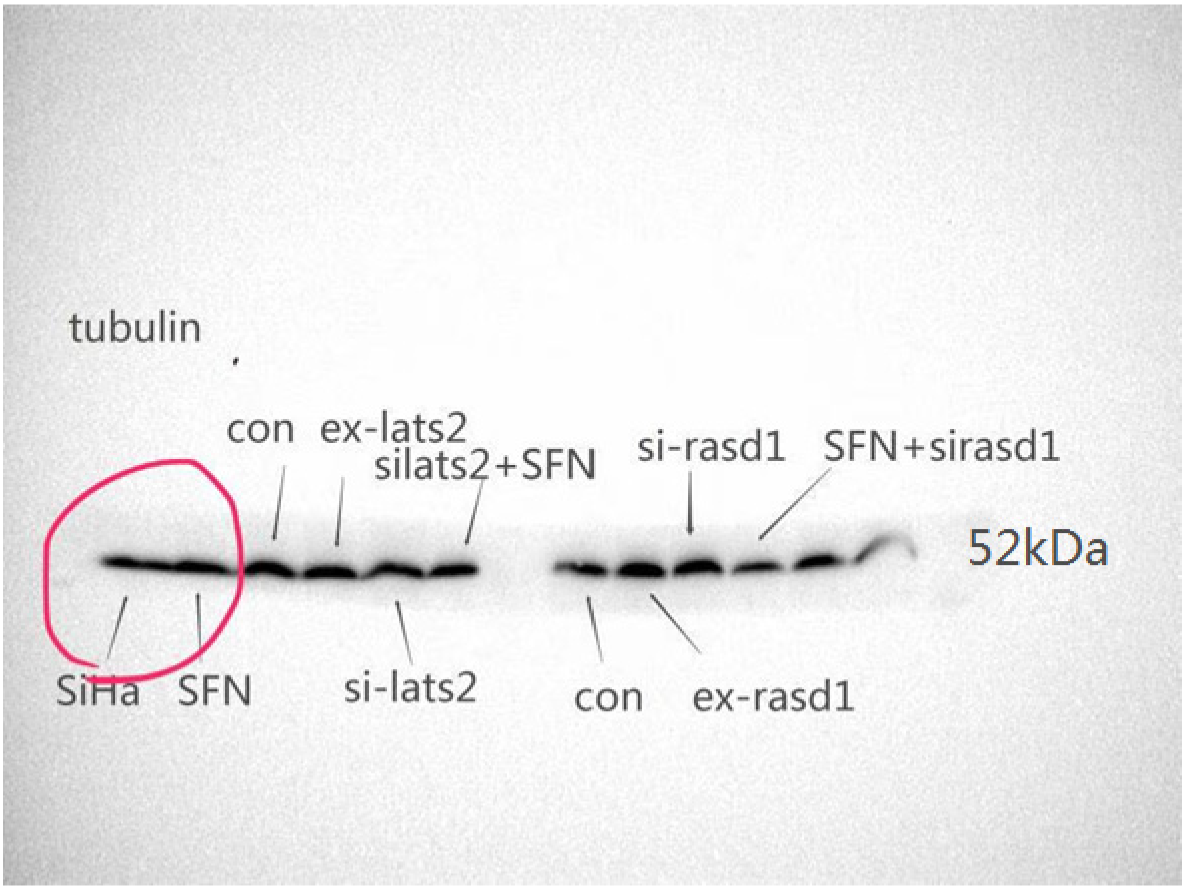

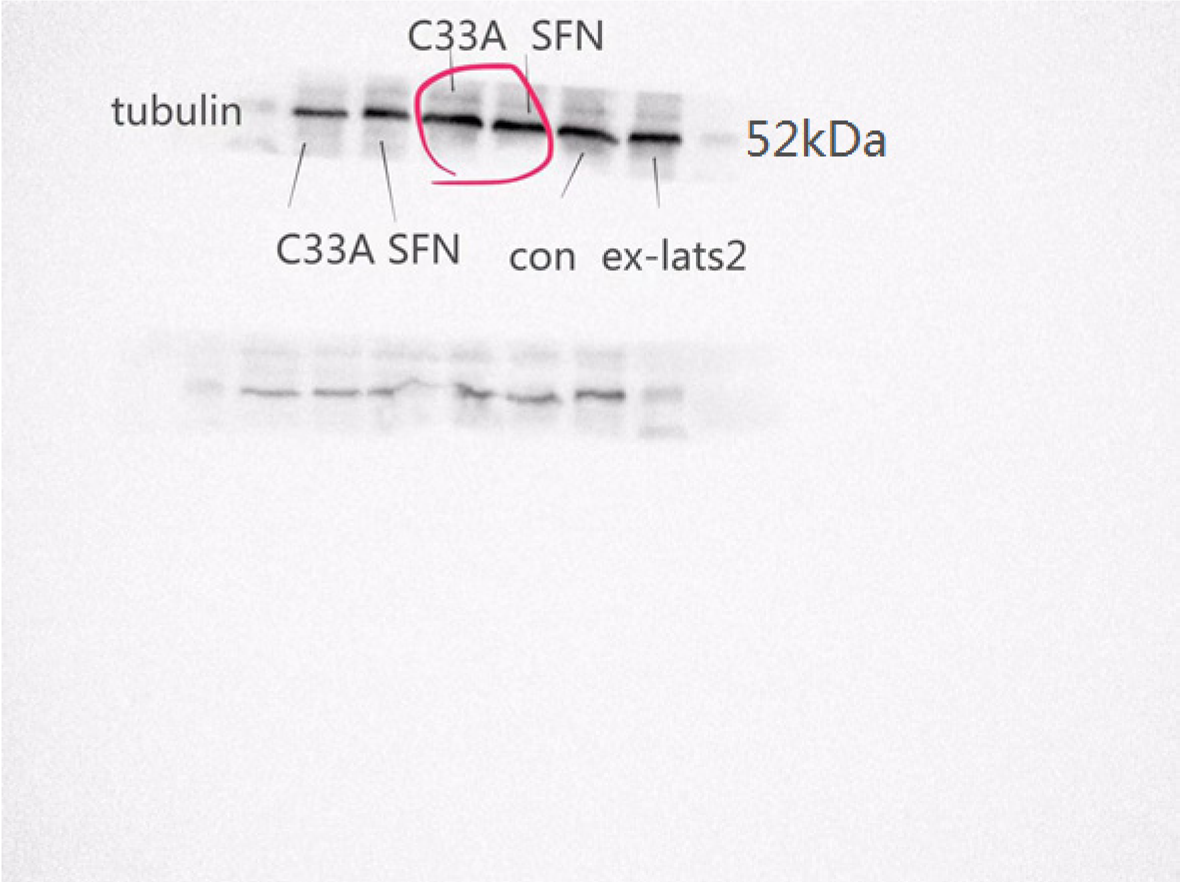

Figure S3. The original western blots for figure 3D.

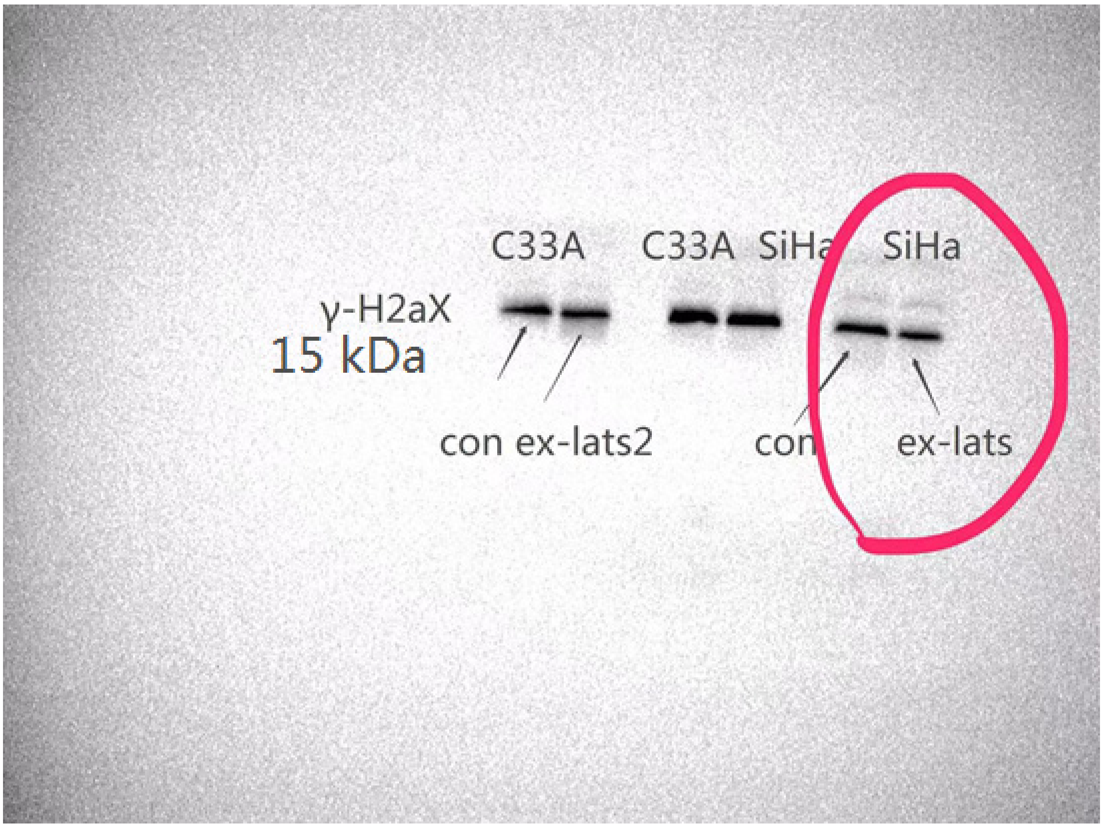
C33A
C33A
SiHa
SiHa
γ-H2aX
15 kDa
con ex-lats2
con
ex-lats

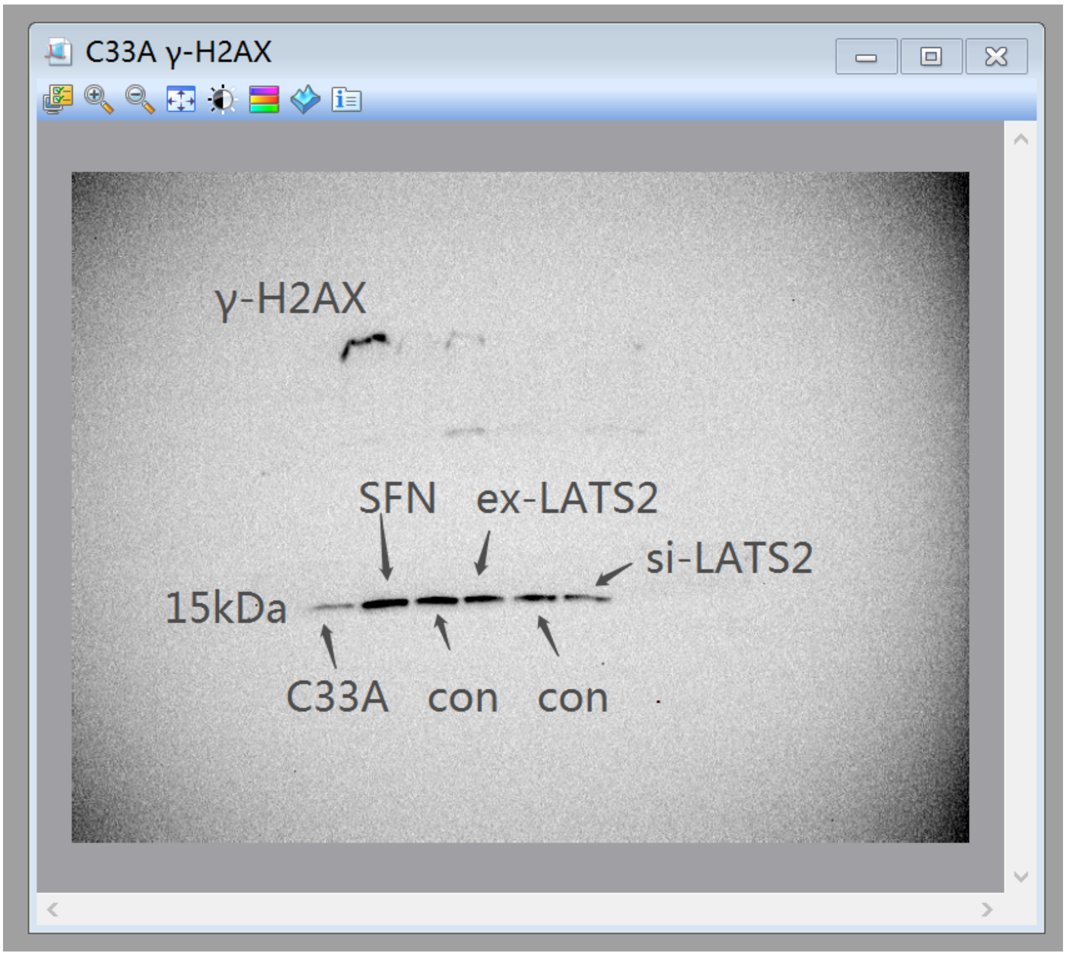
C33A γ-H2AX
γ-H2AX
SFN
ex-LATS2
si-LATS2
15kDa
C33A
con
con

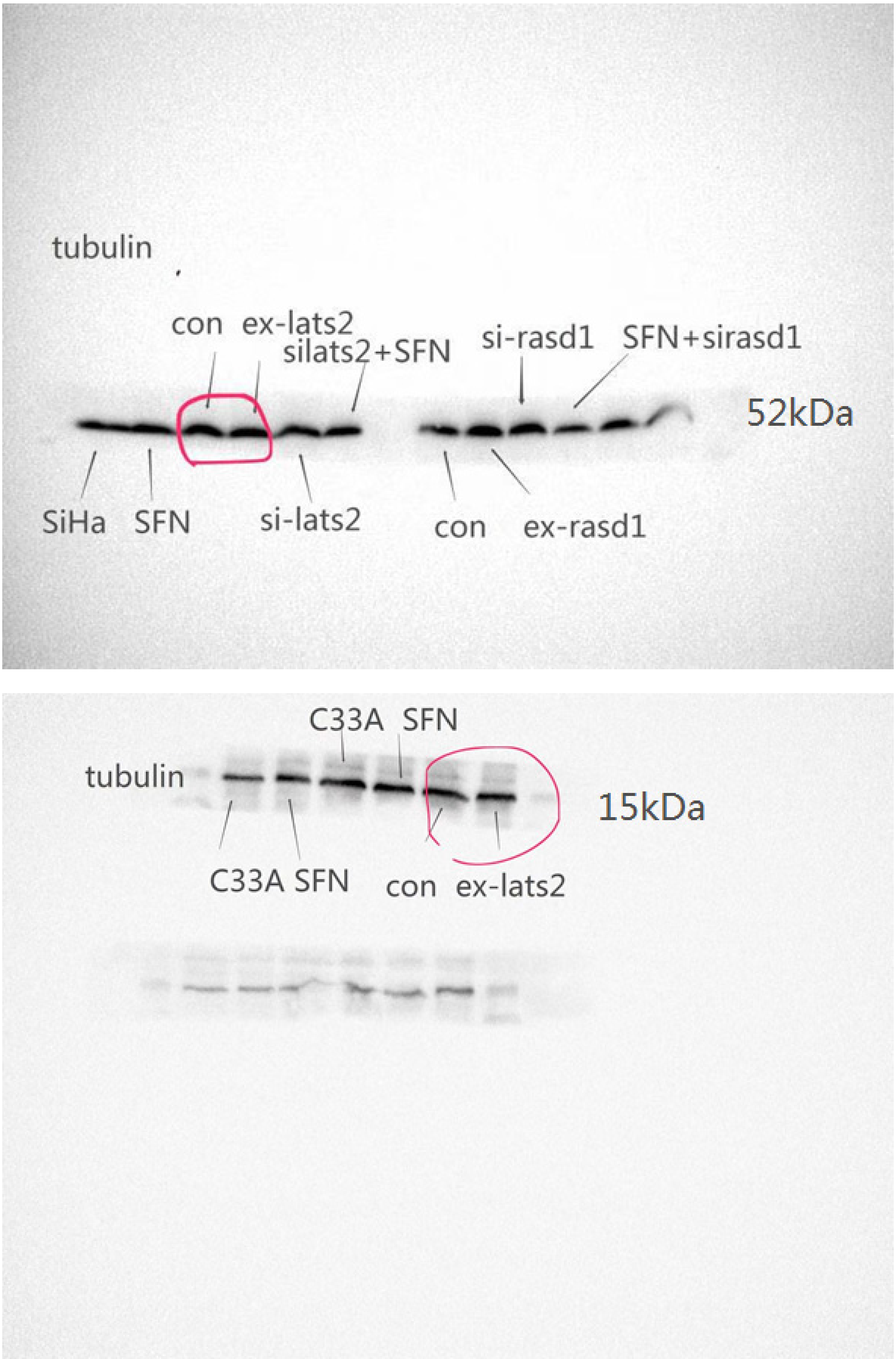

Figure S4. The original western blots for figure 4B.
